# Observations on early fungal infections with relevance for replant disease in fine roots of the rose rootstock *Rosa corymbifera* 'Laxa'

**DOI:** 10.1038/s41598-020-79878-8

**Published:** 2020-12-29

**Authors:** G. Grunewaldt-Stöcker, C. Popp, A. Baumann, S. Fricke, M. Menssen, T. Winkelmann, E. Maiss

**Affiliations:** 1 Institute of Horticultural Production Systems, Section Phytomedicine, Leibniz Universität Hannover, Herrenhäuser Str. 2, 30419 Hannover, Germany; 2 Institute of Horticultural Production Systems, Section of Woody Plant and Propagation Physiology, Leibniz Universität Hannover, Herrenhäuser Str. 2, 30419 Hannover, Germany; 3 Department of Biostatistics, Institute of Cell Biology and Biophysics, Leibniz Universität Hannover, Herrenhäuser Str. 2, 30419 Hannover, Germany

**Keywords:** Microbiology, Plant sciences

## Abstract

Replant disease is a worldwide phenomenon affecting various woody plant genera and species, especially within the Rosaceae. Compared to decades of intensive studies regarding replant disease of apple (ARD), the replant disease of roses (RRD) has hardly been investigated. The etiology of RRD is also still unclear and a remedy desperately needed. In greenhouse pot trials with seedlings of the RRD-sensitive rootstock *Rosa corymbifera* ‘Laxa’ cultured in replant disease affected soils from two different locations, early RRD symptom development was studied in fine roots. In microscopic analyses we found similarities to ARD symptoms with regards to structural damages, impairment in the root hair status, and necroses and blackening in the cortex tissue. Examinations of both whole mounts and thin sections of fine root segments revealed frequent conspicuous fungal infections in association with the cellular disorders. Particularly striking were fungal intracellular structures with pathogenic characteristics that are described for the first time. Isolated fungi from these tissue areas were identified by means of ITS primers, and many of them were members of the Nectriaceae. In a next step, 35 of these isolates were subjected to a multi-locus sequence analysis and the results revealed that several genera and species were involved in the development of RRD within a single rose plant. Inoculations with selected single isolates (*Rugonectria rugulosa* and *Ilyonectria robusta*) in a Perlite assay confirmed their pathogenic relationship to early necrotic host plant reactions, and symptoms were similar to those exhibited in ARD.

## Introduction

Research on apple replant disease (ARD) has gained in importance worldwide, as fruit and young plant cultivations are increasingly affected and the control methods are not yet sufficiently investigated and established to solve the problem completely and sustainably^1–6^. In addition, various trials to select rootstock genotypes and *Malus* germplasm with ARD tolerance are continuously carried out^7–10^. However, the replant disease (RD) is not limited to apple as it also affects related Rosaceous species as well as species of other plant families^11^. In tree nurseries and public parks, Rosaceae shrubs (other than apple) are progressively affected by growth reduction and yield loss if they are continuously cultivated at the same place and in the same soil^12–18^. In *Prunus* spp. like peach, almond, and cherry, prevention and therapy strategies are urgently needed and have been investigated as well^4,13,19–21^. However, studies on rose replant disease (RRD) have rarely been conducted and infection processes of pathogenic fungi have not been documented up to now. The biochemical characterisation of RRD-associated bacteria in the rhizosphere^22^ could neither identify the causal agents of the disease nor offer mitigations. The problem occurred even in cut roses cultured in re-used tuff substrate and was attributed to fungal and Oomyceteous minor pathogens^23^. Since then, there has been little progress in the studies of RRD. However, as early as 1999, Szabo^24^ raised the question of whether the pathogens in apple and rose are identical when RD occurs.

In order to unravel the causal factors of the replant disease, it is necessary to study plant reactions to infectious soil during the first days and weeks of contact. Hoestra^25^, Caruso et al.^26^ and Braun^27^ presented studies with apple plants on this topic. A comprehensive microscopic analysis of first symptoms in fine roots of diverse apple rootstock genotypes led to detailed descriptions of tissue damages and cortex cell reactions. In most cases of cell necroses, fungal infections were microscopically detectable^28^. *Cylindrocarpon*-like fungi (Nectriaceae) have been mentioned repeatedly along with other fungi to be associated with ARD^29–33^. Recently, Manici et al.^34,35^ studied the role of the metabolites of diverse fungal isolates including Nectriaceae species as underestimated components of ARD. Following the isolation of fungal root endophytes from ARD-diseased apple roots, the pathogenicity of several Nectriaceae isolates has been verified in a recently developed Perlite-based inoculation assay^36^. Baumann et al.^37^ presented a greenhouse pot bio-test with seedlings of the RRD-sensitive rose rootstock *Rosa corymbifera* 'Laxa' cultured in RRD-affected soils from two locations differing in soil texture and cropping history.

The objectives of this study, which used plant materials from greenhouse bio-tests of Baumann et al.^37^ and own bio-test materials, were to 1. isolate endophytic root fungi from roots of diseased rose plants, 2. document the early microscopic and histological symptoms in rose fine roots in order to develop diagnostic tools, 3. identify fungal isolates from necrotic tissue of replant diseased roots with molecular biological tools, and 4. evaluate the effects of the identified associated pathogenic fungi in inoculation assays.

## Material and methods

### Rose replant disease bio-tests

In experiment 1, seedlings of *Rosa corymbifera* 'Laxa' served as a source for microscopic analysis and endophytic fungal isolates. In 2017, greenhouse pot trials with roses were conducted with two soils differing in their physical and chemical soil properties: (1) a sandy soil from an experimental area in Heidgraben (H) in the district of Pinneberg, with a 5th replanting of *R. corymbifera* 'Laxa'^38^) and (2) a loamy soil from the Rosarium Sangerhausen (S), an excavation from beds with severe RRD symptoms. In this experiment, the soils were filled into 1 L-containers (ut = untreated soil) with 2 g L^−1^ of the long-term fertiliser Osmocote exact 3–4 M (16 + 9 + 12 + 2MgO + TE; ICL Speciality Fertilizers; ICL Group 2019, https://icl-sf.com/de-de/products/ornamental_horticulture/8840-osmocote-exact-standard-3-4m). One 4-week-old seedling of *R*. *corymbifera* 'Laxa' was planted into each pot (shoot length approx. 3.0 cm). Gamma-irradiated soil samples (treated with a minimum dose of 10 kGy) from each site served as controls and are denoted as γ treatment in the experiments. Twenty replicates per treatment were prepared. Details on this setup as well as plant growth data and their statistical analyses are given in Baumann et al.^37^. After nine weeks of cultivation, roots from five randomly chosen plants per treatment were harvested for isolating fungal endophytes. Four further random plants were examined microscopically. Symptoms in fine roots were analysed by using the same parameters of tissue damages and cortex cell reactions as have recently been reported for apple rootstocks^28^. Prominent fungal infection sites were selected for the isolation of symptom-associated fungi.

In experiment 2, rose seedlings grown in Heidgraben soil (untreated and γ-irradiated, respectively) were used after three weeks of cultivation for microscopic assessment. Here, six pots per treatment were cultured with three plants per pot to save RRD soil. Six randomly harvested root systems per treatment were prepared and analysed using the same parameters as mentioned for plants from experiment 1.

### Isolation of fungal root endophytes from experiment 1

The roots of five plants per treatment (H ut, H γ and S ut, S γ) were gently freed from adhering soil by washing with tap water. To isolate fungal endophytes, roots were surface disinfected: 30 s in 70% ethanol, 2.5 min in 2% NaOCl (with a droplet of Tween 20) followed by washing 5 times in autoclaved distilled water. Twelve 1 cm-root segments per plant were plated on 2% malt extract agar (MEA) amended with oxytetracycline (OTC, 50 µg mL^-1^). Plates were cultured for 2 to 7 days at 24 °C in the dark. Growing mycelium was separated and sub-cultured several times to obtain pure cultures. For identification, isolates were subjected to a direct PCR with universal primers ITS 1 and 4^39^. PCR and sequencing were performed as described in Popp et al.^36^.

During microscopic assessments of fresh unfixed fine roots of *R*. *corymbifera* 'Laxa', more than 120 selected root segments of 1 mm length bearing fungal infection sites of interest were prepared. They originated from three plants of the treatment H ut, which were harvested after nine weeks of cultivation. Root segments were cultured on MEA as described above and isolated fungi identified by ITS PCR. From 90 pure cultures, 66 were selected for further examination, excluding apparent *Trichoderma* and *Penicillium* spp.. Pure cultures of obvious oomycetes (18 isolates) were tested with ITS 6^40^ and ITS 4 as well as primer pairs UN-up18S42^41^ and UN-lo28S22^42^ for their identity. In a second deeper analysis, primer pairs allowing a more specific classification of Nectriaceae were applied to 35 of the isolates: CYLH3F and CYLH3R^43^ for histone H3 (HIS), T1^44^, and Bt-2b^45^ targeting a partial β-tubulin (TUB) gene and CylEF-1 (5′-ATG GGT AAG GAV GAV AAG AC-3′; J.Z. Groenewald, unpublished), together with CylEF-R2^43^ for translation elongation factor 1-α gene (TEF). Sanger sequencing of PCR products was carried out by Microsynth Seqlab (Göttingen, Germany) using the sense primer for each amplification. Results were submitted to BLASTn analysis (Megablast, NCBI, Rockville Pike, USA) and are presented as first hit (sorted by max. score). Isolates were named with the results gained by HIS gene amplification. Accession numbers are given in Supplement Table 1 for ITS analysis and in Supplement Table 2 for representative sequences of Nectriaceae species.

### Microscopy and histology

Sample preparation from fresh fine roots of *R. corymbifera* ‘Laxa’ seedlings and staining with toluidine blue or FUN1 cell stain for microscopic assessments were carried out according to Grunewaldt-Stöcker et al.^28^. In experiment 1, the roots of four randomly chosen plants per treatment were gently washed out and 1 cm-segments of the 1st to 3rd order were randomly sampled and stained with the fluorochrome FUN 1 Cell stain (Molecular Probes, Thermo Fisher Scientific, Life Technologies, USA). Thirty segments per plant were evaluated as whole mounts using brightfield-DIC-microscopy 200–1000×, and epifluorescence microscopy (Zeiss Axio Imager A2, extinction BP 485/20, beam splitter FT 395, emission LP 420). The microscopic observations concerned the parameters damage of the root surface structure, cellular damages and cell vitality in the cortex tissue of the fine root segments, which were recorded as frequencies in rating classes per segment. In addition, the evaluation of the presence of root hairs was divided into four rating classes: many, medium, few, and none, whereas the cell vitality was rated in five percentage classes representing 0, ≤ 25, 26–50, 51–75, > 75% of vital cells per 1 cm-root segment. In experiment 2, fine roots from six plants were harvested after only three weeks of culture and evaluated in the same way, i.e. with 180 random root segments per treatment. The data from both experiments were statistically analysed.

For histological investigations of the thin sections of root tissue, samples from four plants of the variants in Heidgraben soil (H ut and H γ, experiment 1, harvested after nine weeks) were fixed in ethanol formaldehyde glacial acetic acid (AFE^46^). An embedding in glycol methacrylate (GMA^47^) was followed by microtome cutting into thin sections (5 µm; Reichert-Jung, Autocut 2040). After staining of the sections on slides with Toluidine Blue O (C.I. 52,040; Merck) for 1 min and destaining with distilled water, Entellan (Sigma Aldrich, Taufkirchen, Germany) and cover slips were applied for preservation. Four to five tissue sections per slide and three to four slides per embedded block were evaluated. Approximately three to six root samples were cut per block. In total, the number of histological samples examined for variant H ut were ≥ 320 and for variant H γ ≥ 160. The focus of the investigations was on fungal infection structures, host cell reactions, and other endophytes. Photo documentation was performed in all microscopic investigations with a Zeiss Axiocam MRC, AxioVision Release 4.8.

### Statistical analyses

Statistical analyses of data microscopically gained in experiments 1 and 2 were performed using R version 3.6.1 (R Core Team 2019, https://www.R-project.org/). Data management and plotting was done using the tidyverse packages^48^. For each of the parameters observed from experiment 1 the data sets were split into subsets for each of the symptom classes. Each of these subsets was comprised of counted events out of the 30 root segments per plant. Consequently, these counts were assumed to be generated by a binomial process, but exhibit extra binomial variation (also called overdispersion). Therefore, the quasi-likelihood approach^49,50^ was used for modeling. To each subset, a Bayesian Generalized Linear Model running on the logit link was fit in order to stabilize the estimation process in the case that all observations are zero. Only the average proportions depending on the four interaction terms between the soil origin (Heidgraben or Sangerhausen) and the treatment (untreated or γ-irradiated) were modeled. For that purpose, the package arm of Gelmann and Su^51^ was utilized. Ninety-five percent confidence intervals for the model-based average proportions were calculated using the emmeans package provided by Lenth^52^.

Taking the correlations between the symptom classes into account, contrast tests between the modeled average proportions were performed using the Multiple Marginal Model approach^53^ implemented in the multcomp package^54^. For each symptom class, tests (α = 0.05) were calculated between the mean proportions for "H ut and H γ", "S ut and S γ", “H ut and S ut”, "H γ and S γ". The statistical analysis of experiment 2 was similar to that of experiment 1, except that the modeling was only based on the treatments ut and γ. Therefore, the differences between the two treatments were tested in each of the symptom classes.

### Perlite assay with M26 plants

The replant disease susceptible apple rootstock ‘M26′ (hereinafter given as M26) was used in a Perlite assay as indicator plant for the early detection of replant disease symptoms and fungal infections. Micropropagation and acclimatization of M26 plantlets were carried out as described by Mahnkopp et al.^38^. Acclimatized M26 plantlets were cultivated for six weeks under greenhouse conditions (growing medium: Vermehrungssubstrat SP VM; white peat, moderately decomposed (H3–H5), natural clay; organic substance 55%, pH 5.8, salt content 1.5 g L^−1^, N 180 mg L^−1^; P_3_O_2_ 200 mg L^−1^; K_2_O 240 mg L^−1^; S 130 mg L^−1^; MgO 150 mg L^−1^; Balster Einheitserdewerk GmbH, Fröndenberg, Germany). Perlite assays were performed according to Popp et al.^36^ with M26 plantlets each in one Petri dish filled with fungal inoculum pre-cultured in a mixture of Perlite and malt extract. Control plants were grown in Perlite plus malt extract without fungal cultures. Eight plants per treatment were tested over a period of up to 51 days. Fungal isolates originating from *R. corymbifera* 'Laxa' (isolated from microscopically selected necrotic tissue areas of root segments, which were harvested of plants of the treatment H ut) were tested for pathogenicity and symptom induction in micropropagated M26 plantlets. Two isolates of *Rugonectria rugulosa* (RRD 26 and RRD 28) as well as two isolates of *Ilyonectria robusta* (RRD 27 and RRD 70) were used for this artificial inoculation. Wilting and dying shoots were assessed without any aids, whereas discolorations, necroses, and fungal infections in the root tissue were analysed microscopically. Fresh fine roots of eight apple M26 plants harvested from the Perlite assay were examined microscopically for fungal infection sites and tissue symptoms as is given above for the samples of *R. corymbifera*. Callose deposits at fungal penetration sites were stained in fresh roots with aniline blue in phosphate buffer (0.005%, pH 8.5)^46^ and examined with epifluorescence (UV filter set, extinction 365, beam splitter FT 510, emission LP 515).

## Results

In experiment 1, the *R. corymbifera* ‘Laxa’ plants had previously been assessed to be severely affected in both shoot and root growth parameters, indicating the presence of RRD in both soils. The significant differences in growth data in untreated versus γ-irradiated soil presented by Baumann et al.^37^ confirmed the RRD status of the experimental plants from which random samples were used for our purposes. The difference in plant appearance for the two soil variants is exemplarily illustrated for soils from the Heidgraben site (Fig. ESM 1a,b).

### Isolation of endophytic root fungi from experiment 1

**Figure 1:**
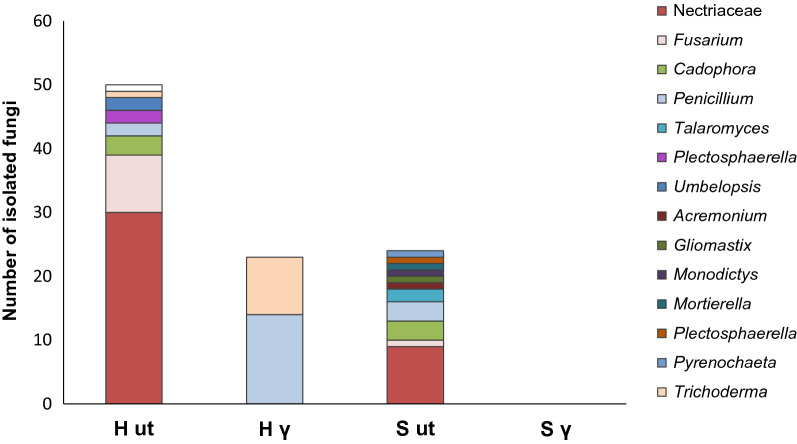
Fungal root endophytes isolated from surface disinfected roots of five *R. corymbifera* 'Laxa' plants that were grown for 9 weeks in untreated (ut) or irradiated (γ) soils from replant diseased sites Heidgraben (H) and Sangerhausen (S), respectively (experiment 1). Number of isolates gained from 60 root segments per treatment, identified by ITS PCR and Sanger sequencing, and presented as family (Nectriaceae, exclusive *Fusarium*) and as genus.

In a first attempt to identify endophytic fungi in roots of the replant diseased plants, a wide range of species was isolated from roots grown in untreated soils, and fewer (H γ) or no (S γ) isolates were gained from roots cultivated in irradiated soils (Fig. 1). Isolates identified by ITS primers 1 and 4 as members of Nectriaceae (belonging to the genera *Calonectria, Cyclindrocladiella*, *Dactylonectria, Ilyonectria, Nectria*) were most prominent in untreated soil treatments. *Cadophora* spp., *Fusarium* spp., and *Penicillium* spp. were present in smaller proportions. From roots of plants grown in irradiated soils, only *Penicillium* and *Trichoderma* isolates were obtained from the Heidgraben treatment (H γ) (Supplement Table 2, F isolates).

### Microscopic analysis of rose fine roots in whole mounts

**Figure 2:**
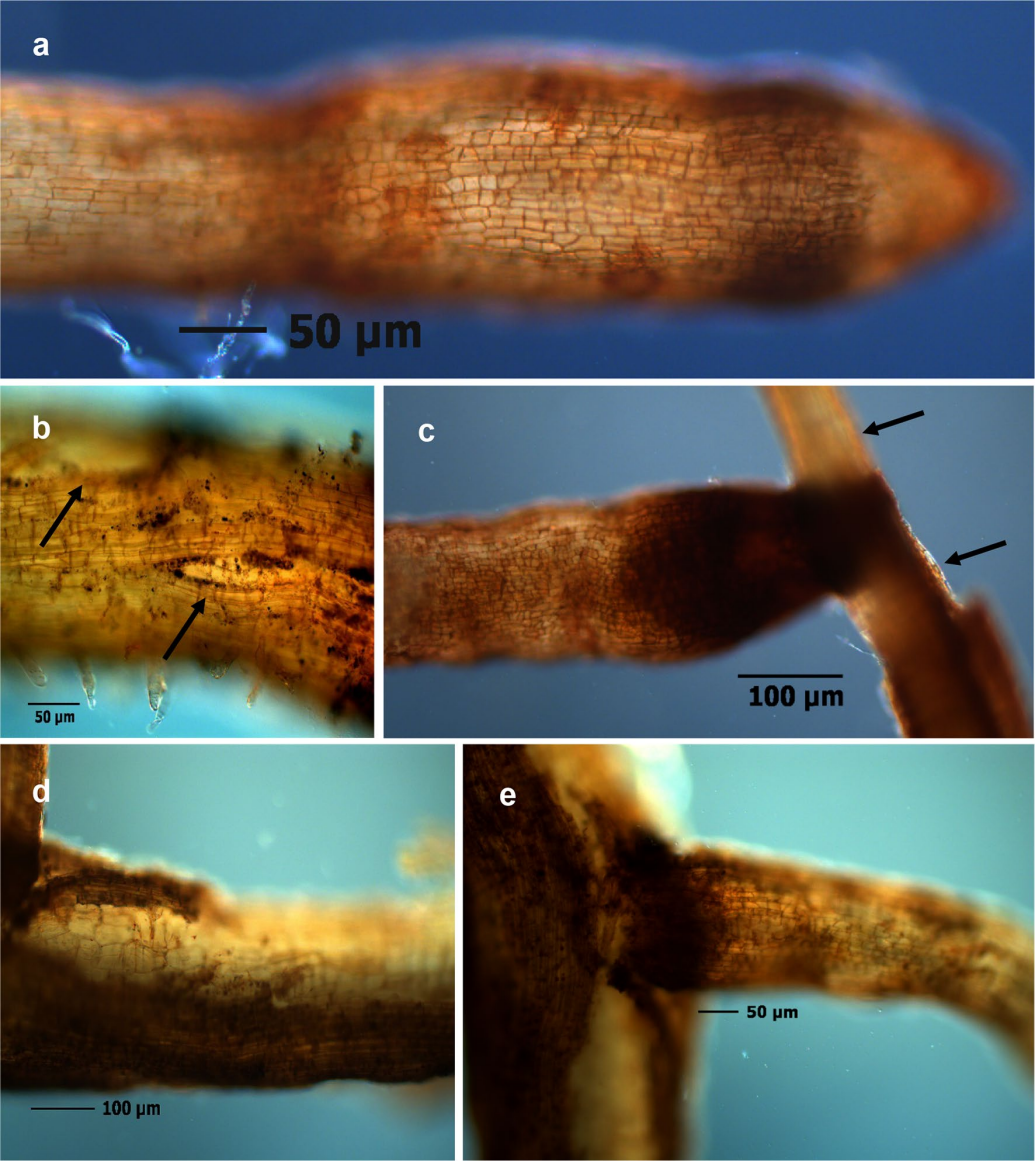
Structural damages of outer tissue layers in fine roots of *R. corymbifera* ‘Laxa’, 9 weeks after cultivation in replant diseased soils from site Heidgraben (**a**,**b**,**d**) and site Sangerhausen (**c**,**e**). Constrictions in newly formed roots (**a**), ruptures in and loss of cortex tissue (arrows) next to branching lateral roots (**b**–**e**).

**Figure 3:**
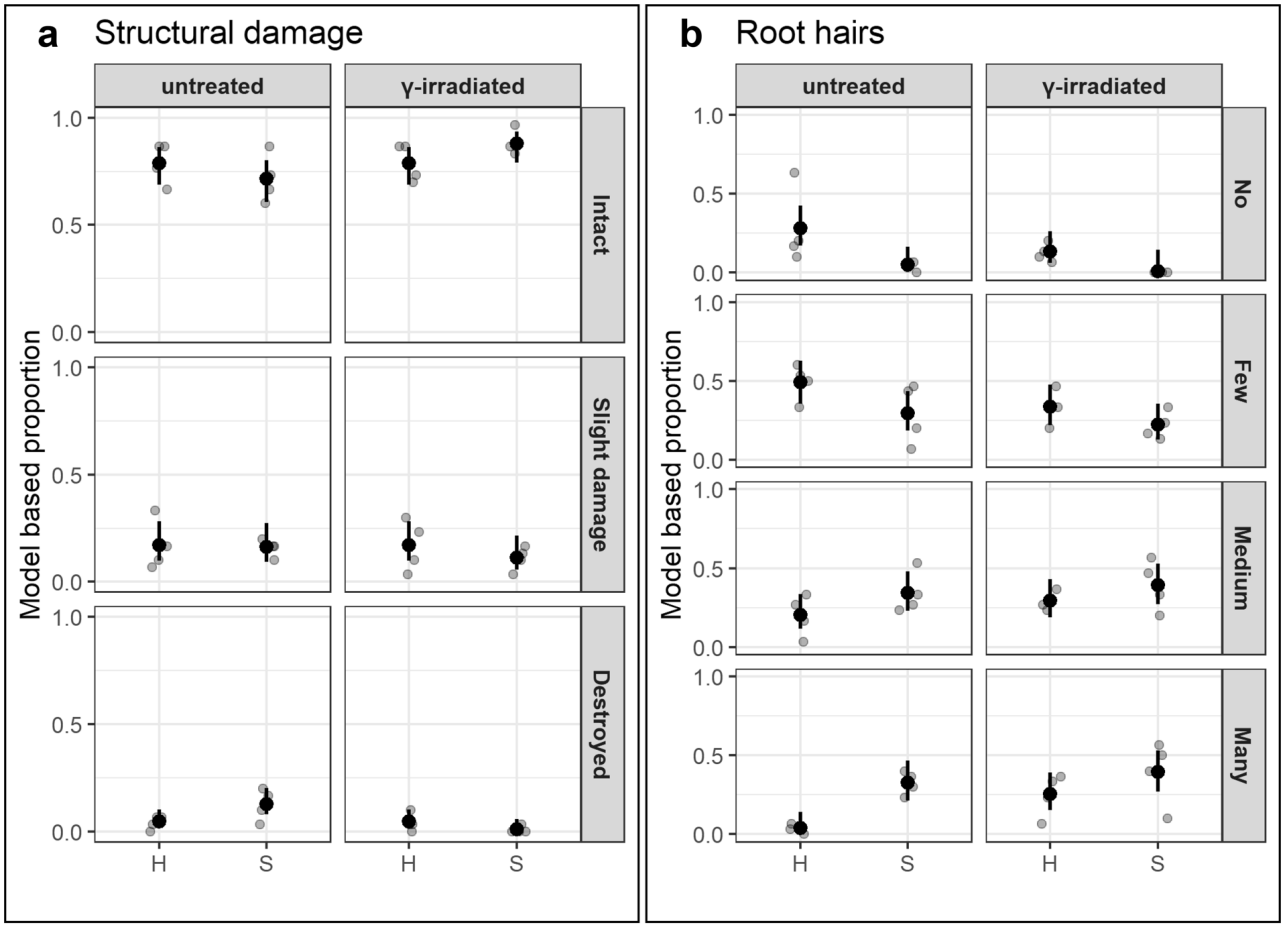
Proportions of structural damages in the outer root layers (**a**), and of the root hair status (**b**) in replant diseased fine roots of *R. corymbifera* ‘Laxa’, after 9 weeks of cultivation in untreated or γ-irradiated RRD soil from Heidgraben (H) in comparison to fine roots grown in RRD soil from Sangerhausen (S). Assessments separated into three (structural damage) and four symptom classes (root hairs), respectively, are given as observed proportions per plant (grey dots), mean proportions driven from the models (black dots), and 95% confidence intervals for the mean proportions (black bars) from 30 root segments of n = 4 plants. Significant difference (α = 0.05) were found in (**a**) for “destroyed” Sangerhausen untreated vs. Sangerhausen γ-irradiated (p = 0.0354) and in (**b**) for “many root hairs” Heidgraben untreated vs. Sangerhausen untreated (p = 0.0177). This figure was created using the software R version 3.6.1 (R Core Team 2019, https://www.R-project.org/)^48,51,52,54^.

The randomly selected root segments from experiment 1 evenly represented roots of 1^st^ (with tip), 2^nd^, and 3^rd^ order (Fig. ESM 2). Structural damage occurred as constrictions in lateral roots, ruptured cortex, and loss of cortex cell layers (Fig. 2a–e). Fine roots from untreated soils of the Sangerhausen site were significantly more damaged in the outer structure, class “destroyed”, than those from irradiated soil; this effect was less clearly expressed in the roots from Heidgraben treatments (Fig. 3a). The status of root hairs differed significantly between the treatments H ut and S ut in the rating class "many", indicating a stronger negative effect of soil of Heidgraben as compared to soil of Sangerhausen (Fig. 3b).

**Figure 4:**
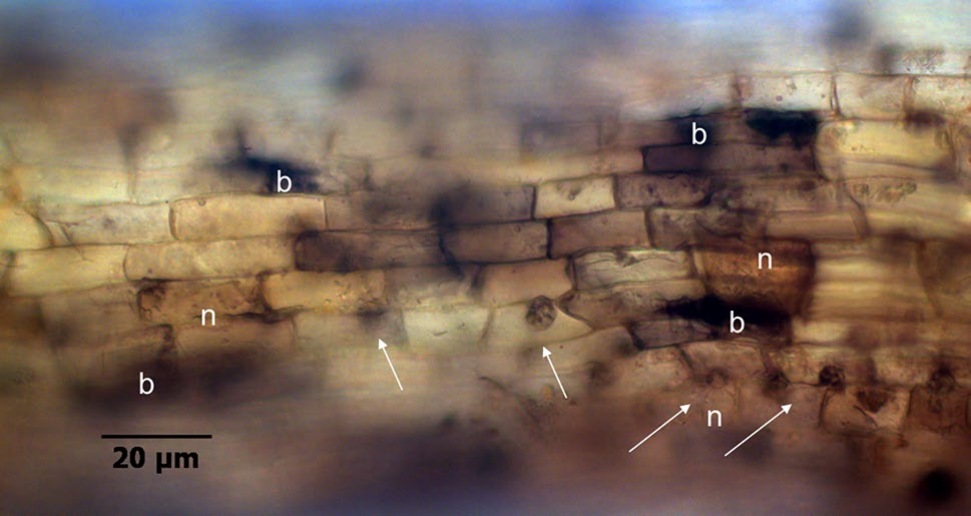
Cell necroses (n), blackening (b), and dark fungal structures in rhizodermal cells (arrows) in a fine root of *R. corymbifera* ‘Laxa’, after 9 weeks of cultivation in RRD soil from site Heidgraben.

Cellular damages, especially necrosis and blackening, were visible when affected cells formed brown and later black clusters in the rhizodermal tissue (Fig. 4). Necrotic tissue occurred in 30% of all root samples from untreated soils, which was twice as much as in roots from irradiated soils, when all reads of four plants per treatment are considered together. Cytoplasmic inclusion bodies were frequently found, but almost exclusively in roots from Heidgraben soil (H ut). Light browning occurred in a large proportion of roots in untreated as well as in irradiated soils and is thus to be excluded from RD characteristics. Further, disorders of the cell function were made visible by specific vitality staining in the rhizodermal and hypodermal cell layers. Compared to plants grown in irradiated RRD soils, there was a tendency in untreated soils of both origins for a negative impact on the metabolic activity in fine roots with less vital cells per segment. More cells were disintegrated or even necrotic, and only a few and scattered vital cells with green nuclei and red intravacuolar stain particles remained (Fig. ESM 3a–c). However, these differences in the occurrence of cell damages and cell vitality, which are based on values of four plants, were not statistically significant (Fig. ESM 4a,b).

**Figure 5:**
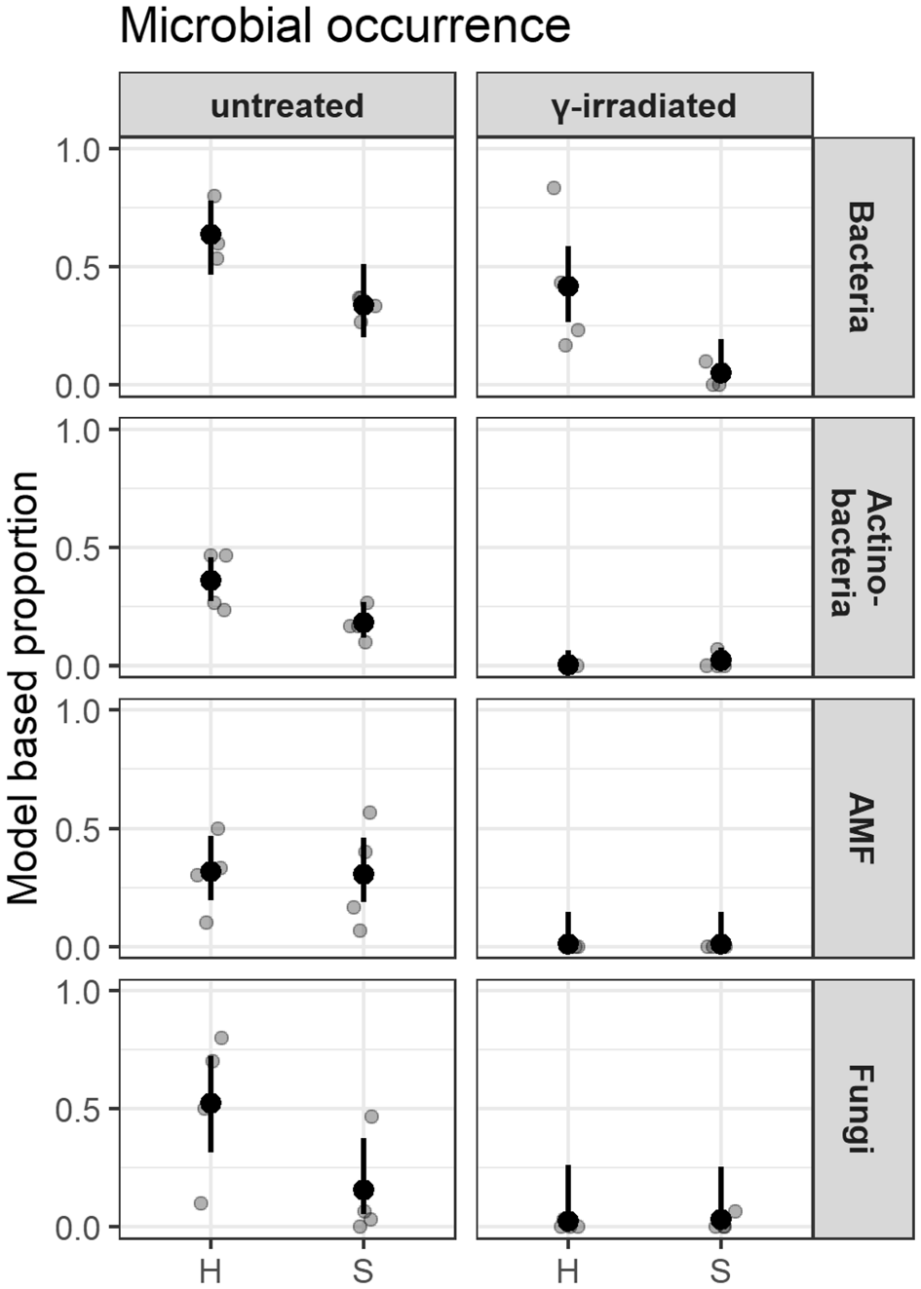
Occurrence of microorganisms in fine roots of *R. corymbifera* ‘Laxa’ grown in untreated or γ-irradiated soil from replant diseased sites Heidgraben (H) and Sangerhausen (S), respectively. Proportions of root segments rated for bacteria, actinobacteria, arbuscular mycorrhiza fungi (AMF), and other fungi. Given are observed proportions per plant (grey dots), mean proportions driven from the models (black dots), and 95% confidence intervals for the mean proportions (black bars) from 30 root segments of n = 4 plants. Significant differences (α = 0.05) were found for the occurrence of bacteria in Heidgraben γ-irradiated vs. Sangerhausen γ-irradiated (*p* = 0.027) and for actinobacteria in Heidgraben untreated vs. Heidgraben γ-irradiated (*p* < 0.01), as well as for actinobacteria in Sangerhausen untreated vs. Sangerhausen γ-irradiated (*p* = 0.016). This figure was created using the software R version 3.6.1 (R Core Team 2019, https://www.R-project.org/)^48,51,52,54^.

**Figure 6:**
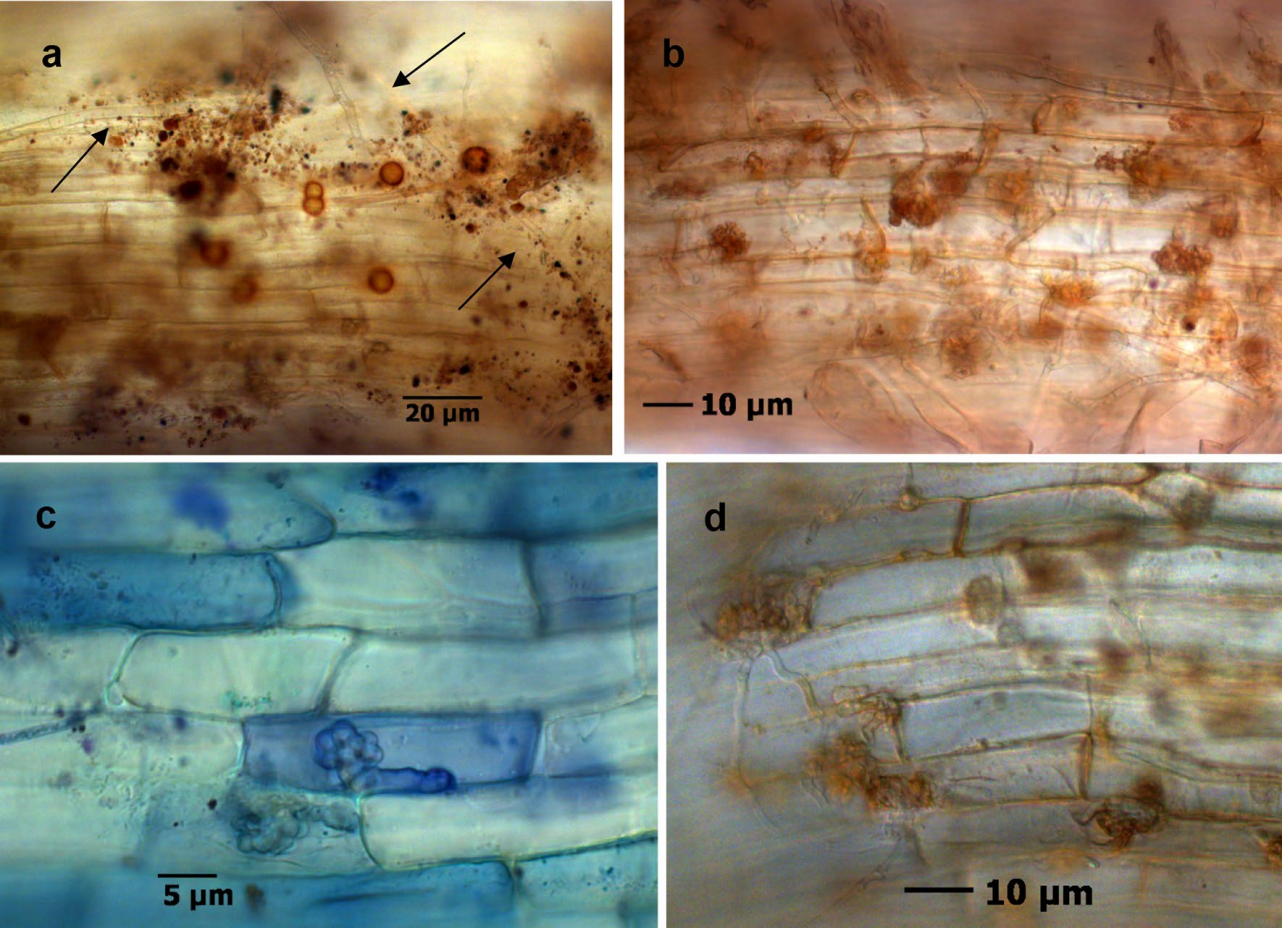
Fresh fine roots of *R. corymbifera* ‘Laxa’ affected by replant disease in soil from Heidgraben after three weeks of cultivation, harbouring CF structures forming fungi: Septated crude mycelium (arrows) and extraradical brown chlamydospores (**a**), intracellular CF structures and fungal hyphae in rhizodermal cells (**b**), and intracellular hyphae and CF structures in disintegrated cells (**c** with toluidine blue stain, **d** with FUN®1 cell stain).

Bacteria and actinobacteria were detected by fluorescence microscopy and assessed in frequencies per root segments. Roots in untreated soils of Heidgraben and Sangerhausen, respectively, were colonised by actinobacteria to a significant greater extent than those in the corresponding irradiated soils. Fungi and rarely oomycetes were present in damaged tissue of roots in untreated soils, whereas the occurrence of fungi was low in roots grown in irradiated soils (Fig. 5). Some of the fungi could be classified morphologically as *Nectria*-like due to their chlamydospores. In high frequencies, intracellular condense nodular fungal structures with the shape of a cauliflower head (CF) occurred in infected root areas (Fig. 6a–d) and were observed in both treatments, H ut and S ut. In fine root samples of replant diseased plants from experiment 2, these CF structures were also found as early as three weeks after cultivation in untreated Heidgraben soil.

**Figure 7:**
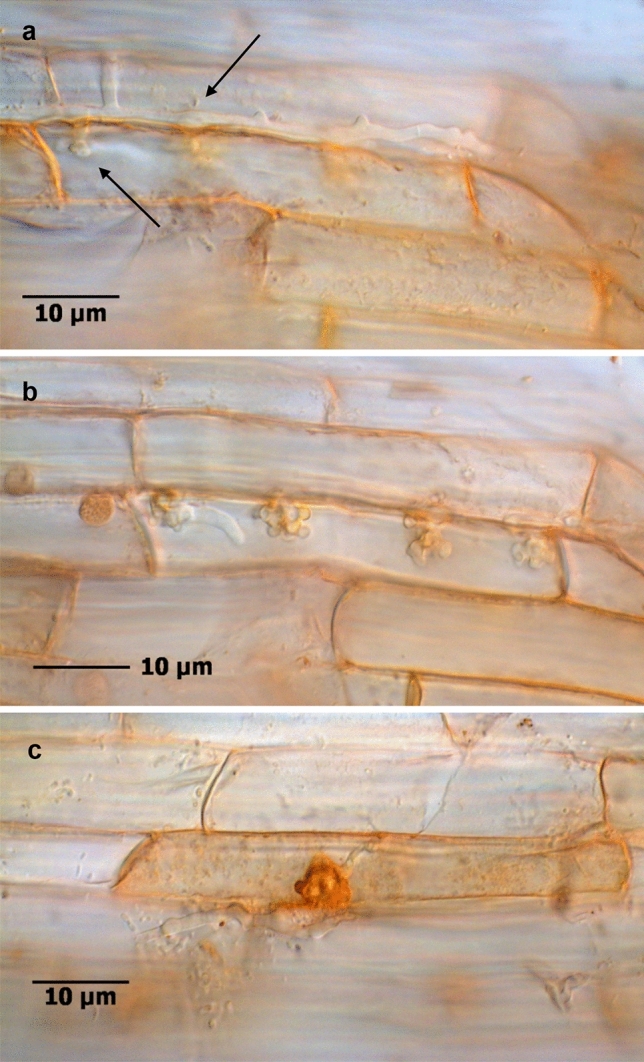
Fine root of *R. corymbifera* ‘Laxa’ with the infection of a CF structure forming fungus. Intraradical hyphae and cell penetration (**a**, arrows), four intracellular CF structures and a connected growing hypha (**b**), and cell necrosis during penetration and CF formation (**c**). FUN®1 cell stain, bright field microscopy.

Such infections started with the direct penetration of a strong mycelium either into a rhizodermal cell or into the apoplast at cellular junctions. The compact CF structures formed inside the host cell and turned brownish, later black. The infection process led to the impairment of host cells with disintegration of cell components, browning, necrotic collapse (Fig. 7a–c), and blackening of whole cells, ultimately extending to neighbouring cells. Further, occasionally brown to black intracellular inclusion bodies of different size were detected in infected areas.

Another fungus with impressive production of melanised arthroconidia on the root surface developed rough mycelial structures within the outer cell layers of fine roots of two plants (treatment H ut) (Fig. ESM 5a,b). Therefore, it is classified as an endophytic fungus; however, its identity has yet to be determined.

After nine weeks of cultivation, mycorrhiza colonisation was easily detected with FUN1 cell staining due to fluorescent arbuscular structures in the cortex cells (Fig. ESM 6). In the brightfield analysis, typical appressoria, distinct massive penetration sites, and intracellular coiled hyphae in the first cortex cell layers were visible, but only in healthy appearing root segments grown in untreated soils from Heidgraben as well as from Sangerhausen.

Dark septate endophytes (DSE) occasionally colonised fine roots of untreated variants (H ut and S ut, respectively) and developed microsclerotic cell groups (Fig. ESM 7). Curved septate macroconidia with the appearance of those belonging to *Fusarium oxysporum* and *F. solani* were not visually detectable, but the isolation of these pathogens from tissue with CF structures implies their existence in replant diseased fine roots that had been grown in RRD-affected soil. Oomycetes rarely appeared in tissues; they were identified because of their thick-walled oospores.

Summarising the site effect of the soil variants used: Plants grown in soil from Sangerhausen (S ut) exhibited less pronounced RRD root symptoms than those from Heidgraben soil (H ut) with respect to the distinct impaired formation of root hairs and the bacterial colonisation of rose fine roots. There was no difference between the two untreated soils (H ut vs. S ut) regarding cell damages and discoloration, cell vitality and structural damage in the fine roots after nine weeks of culture. In both irradiated soils, the microbial load was markedly reduced, except for the frequency of bacteria in the Heidgraben variant (H γ).

Regarding the early plant reactions to RRD soils, the microscopic evaluation of fine roots from six plants in experiment 2 with Heidgraben soils showed the beginning of RRD after only three weeks of cultivation in untreated soil. Cell vitality in the outer tissue layers was reduced compared to roots grown in the irradiated soil since more non-vital cells (0% or > 0–25% vitality) were found in untreated than in irradiated soils. Vice versa, the γ-irradiation lead to higher proportions of vital cells (vitality > 25% up to 100%) than in untreated soil (Fig. ESM 8a). A higher percentage of browning in untreated than in γ-irradiated soil was observed, but none of the tests calculated for root damage was significant with *p* = 0.05 (Fig. ESM 8b). The microbial colonisation of fine roots was highly present in untreated soil at this stage and differed significantly from that in irradiated soil for bacteria, actinobacteria, fungi and CF fungi, but not for AMF (Fig. ESM 8c). Mycorrhizal colonization did not occur that early. Especially fungi (as well as bacteria and actinobacteria) were detectable in more than 50% of the root segments, and also fungi forming CF structures and chlamydospores were identified that early.

### Histological studies in thin sections of rose root tissue

In order to gain deeper insights into sites with the intracellular CF fungal structures in necrotic tissue parts, root segments from Heidgraben samples (H ut, H γ) of experiment 1 were analysed in thin sections.

**Figure 8:**
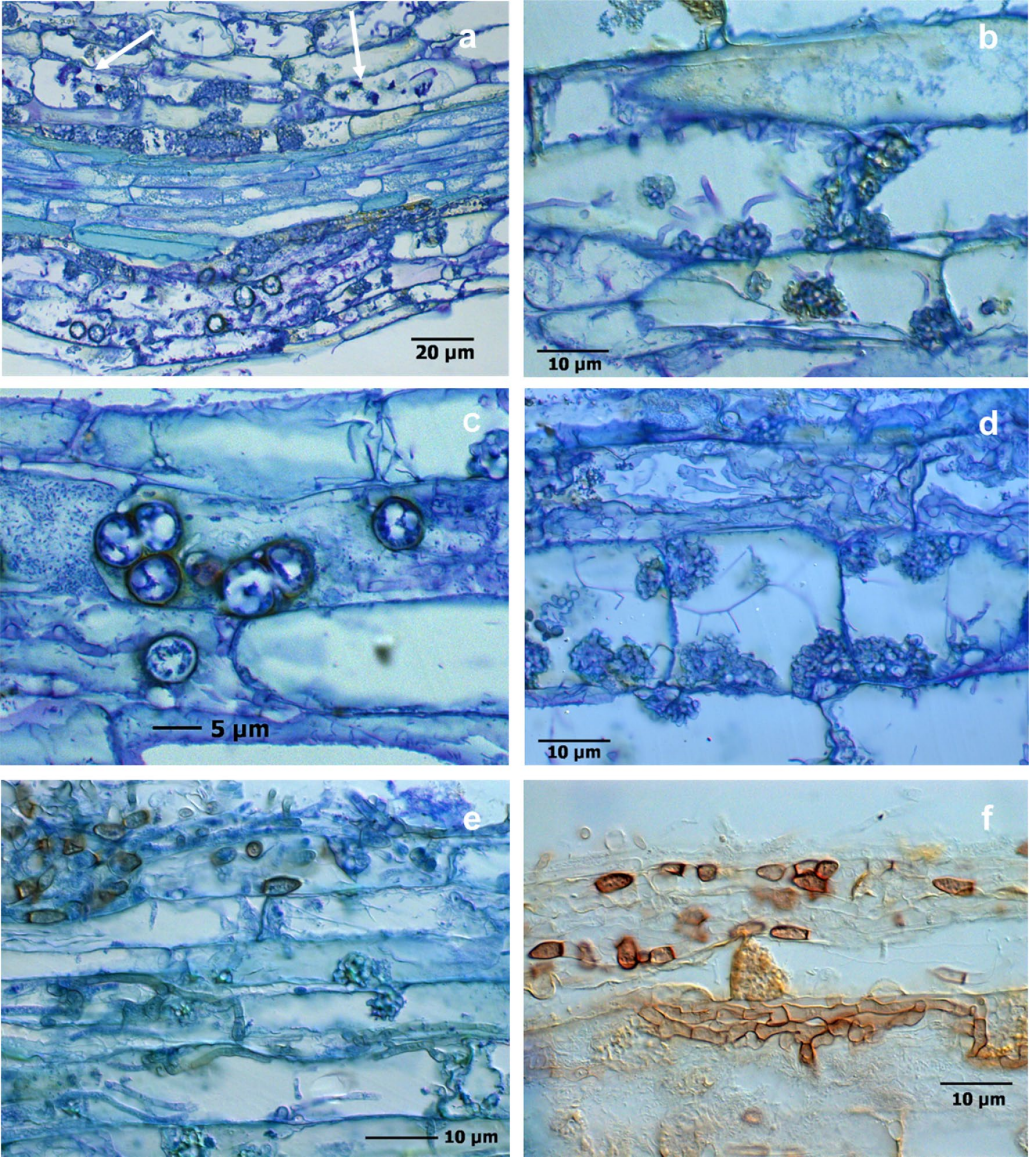
Thin sections of *R. corymbifera* ‘Laxa’ fine roots, after 9 weeks of cultivation in untreated RRD soil from site Heidgraben with fungal infections, toluidine blue stained. Infected cortex cells including CF structures, round shaped chlamydospores, residues of mycorrhizal arbuscules (arrows) and a non-infected stele part (**a**), necrotic cells with CF structures and intracellular hyphae (**b**), cortex cells infected with bacteria and developing chlamydospores (**c**), heavily infected cells with numerous CF structures, thin actinobacterial threads, and intercellular fungal hyphae (**d**), mycelia with arthroconidia on the surface and intracellular mycelia forming CF structures (**e**), and a still unidentified fungus with arthroconidia and short-celled mycelia, unstained (**f**).

The rose roots grown for nine weeks in untreated RD soil from Heidgraben (H ut) showed severe infections by the CF-forming fungus, which extended over the whole cortex up to the endodermis. The endodermis formed an impassable barrier due to which the central cylinder remained free of this type of infection. In some samples, intracellular chlamydospore formation was detected (Fig. 8a,c), intracellular hyphae and CF structures led to the disintegration of host cell components, and ultimately resulted in a cellular collapse and a light brown coloration (Fig. 8b). Further, simultaneous infections with bacteria (Fig. 8c) and actinobacteria (Fig. 8d) affected damaged cells of the rhizodermal layer and cortex parenchyma, mainly in necrotic areas. In the vicinity to CF structures, dense mycelia colonised rhizodermal cells and formed arthroconidia on the root surface, as was found before in total preparations (Fig. 8e,f).

Thin sections also revealed residues of mycorrhizal colonization in infected tissue areas. Degenerated arbuscules and CF structures in adjacent necrotic cortex cells displayed the strength of the pathogens, whereas healthy appearing root segments with undisturbed mycorrhiza establishment rarely occurred in variants from untreated replant diseased soils (S ut, H ut) (Fig. 8a, Fig. ESM 9a).

**Figure 9:**
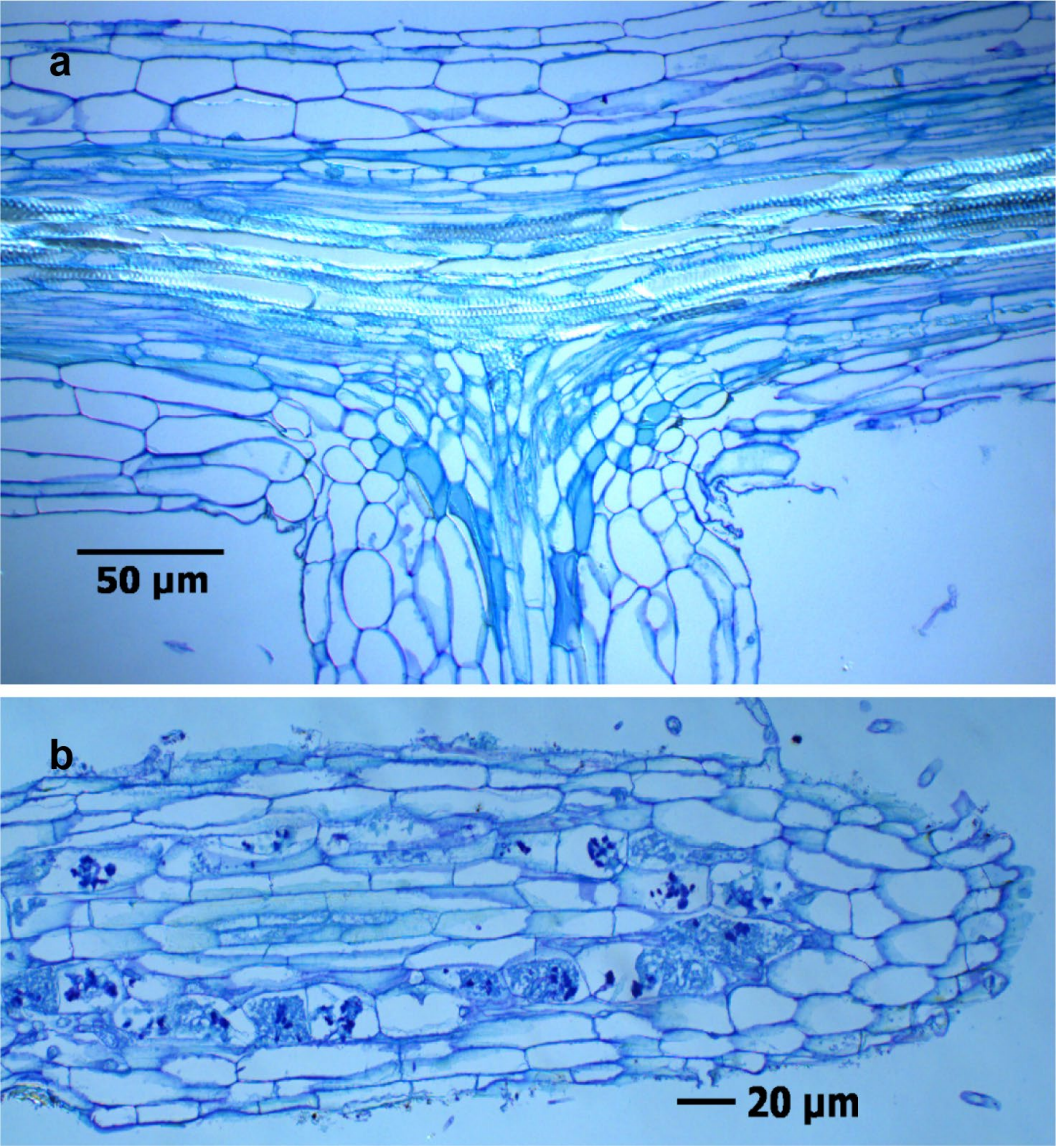
Thin sections of healthy fine root segments of *R. corymbifera* ‘Laxa’ 9 weeks after start of the biotest. Intact tissue with outgoing lateral root (**a**; treatment H γ) and with mycorrhizal colonization of cortex cells (**b**; treatment H ut).

Infestation with endoparasitic nematodes led to increased bacterial infections in the root tissue (Fig. ESM 9b). Almost all root segments colonised by fungi were damaged. Few root segments had tissue areas with structurally intact cells and with evenly arranged mycorrhizal colonisation (Fig. 9a,b). Such healthy tissue was regularly found in root samples from irradiated soil (H γ). Here, neither fungal infections nor colonisation with mycorrhizal fungi occurred in the tissue. The presence of bacteria was limited to the root surface.

### Identification of fungal isolates from necrotic tissue in fine roots of replant diseased plants

During microscopy of fine root segments from rose plants of the experiment 1, three rose plants grown in the untreated Heidgraben RRD soil (H ut) served as origin for endophytic fungal isolates gained from non-disinfected, tiny small root pieces with conspicuous infection sites. Based on colony morphology, colour, and occasionally on spore formation, 66 from more than 90 pure cultures (RRD) were selected for identification in a direct PCR with primers ITS 1 and ITS 4. The subjective selection of agar cultures focused on potential Nectriaceae and some obvious *Fusarium* isolates were included. The aim was to determine the relationship of specific fungal root endophytes to the infection sites described before and not their actual frequencies in the root systems. This first molecular identification assay led to Nectriaceae in the majority of isolates (37 of 66, Supplement Table 1).

Out of eighteen oomycetes additionally isolated from another two rose plants harvested after nine weeks of cultivation, 16 were identified via direct PCR (primers ITS 4 and ITS 6) and also with primer pairs UN-up18S42 and UN-lo28S22 with 100% BLAST identity level as *Globisporangium sylvaticum* (former name *Pythium sylvaticum*). Two isolates belonged to the species *Phytopythium vexans* (former name *Pythium vexans*).

Thirty-five of the RRD Nectriaceae isolates were subjected to a second identification process with three differing primer pairs that confirmed the Nectriaceae, but with partially deviating results when regarding the certainty of the determination of genera and species (Supplement Table 2). Therefore, we named the isolates according to HIS results. *Ilyonectria robusta* (8 isolates)*, Dactylonectria estremocensis* (2 isolates)*, D. pauciseptata* (2 isolates), and *D. torresensis* (6 isolates) were identified with at least three concordant hits. The predominantly defined isolates in ITS analyses (*Nectria* spp. isolates, 15 of 35) were identified as *Rugonectria rugulosa* when determining the HIS gene sequence (14 of 15), and isolate RRD 65 turned out to be *I. robusta.* On the other hand, TUB gene sequencing identified the 14 isolates as *Thelonectria rubrococca* (7) and *Thelonectria* spp. (7), while RRD 65 was again identified as *I. robusta*. Even *Calonectria* sp. (*montana*) and *Cylindrocladiella* sp. (*parva*) were detected in the collection of RRD isolates. Overall, the 35 isolates belonged to eight different species. As far as the original host plants are concerned, two of them harboured several species of different genera. The overall number of isolates of each species (named according to the results of the most effective gene-sequence HIS) from either plant is given in Table 1. Thus, there is a spectrum of Nectriaceae involved in early infections of rose fine roots in this replant diseased soil.

**Table 1:** Summary of identified fungi by the HIS-gene sequence of selected Nectriaceous isolates gained from replant diseased *Rosa corymbifera* ‘Laxa’.

Plant no.	Tested isolates	HIS-identified fungi	Number
1	1	*Rugonectria rugulosa*	1
2	11	*Rugonectria rugulosa*	4
		*Ilyonectria robusta*	3
		*Dactylonectria pauciseptata*	2
		*Dactylonectria hordeicola*	1
		*Calonectria montana*	1
3	23	*Rugonectria rugulosa*	9
		*Dactylonectria torresensis*	6
		*Ilyonectria robusta*	5
		*Dactylonectria estremocensis*	2
		*Cylindrocladiella* spp.	1

### Perlite assay with RRD isolates

It was one aim of this study to reveal the expression of shoot and root symptoms as reaction to infections with single fungal isolates of the Nectriaceae. Here, we report on selected isolates obtained from conspicuous infections sites in a single replant diseased *Rosa corymbifera* ‘Laxa’ plant. Two isolates of *R*. *rugulosa* and two isolates of *I. robusta* (Table 2) were tested.

**Table 2:** Symptom analyses of M26 shoots and roots in a Perlite assay with temporally staggered microscopic root evaluation 4 to 52 dpi, n ≥ 4 plants, summary of symptom occurrence: – no, (+) slight, + prevalent, ++ heavy, + + + strong. *Rugonectria rugulosa* and *Ilyonectria robusta* isolates (RRD) obtained from one plant of *Rosa corymbifera* ‘Laxa’ after cultivation in RRD soil from the Heidgraben site and named according to the HIS gene sequence results.

Isolate	Identity	Shoot wilting	Root blackening	Root necroses	CF structures	Cytoplasmic inclusions
RRD 26	*R. rugulosa*	**(+)**	** + + **	**+ + **	**+ + **	**–**
RRD 28	*R. rugulosa*	** + + **	**–**	**+ **	**+ + + **	**–**
RRD 27	*I. robusta*	** + + + **	**–**	**+ + **	**–**	** + + **
RRD 70	*I. robusta*	** + + + **	**–**	**+ + **	**–**	** + + **

**Figure 10:**
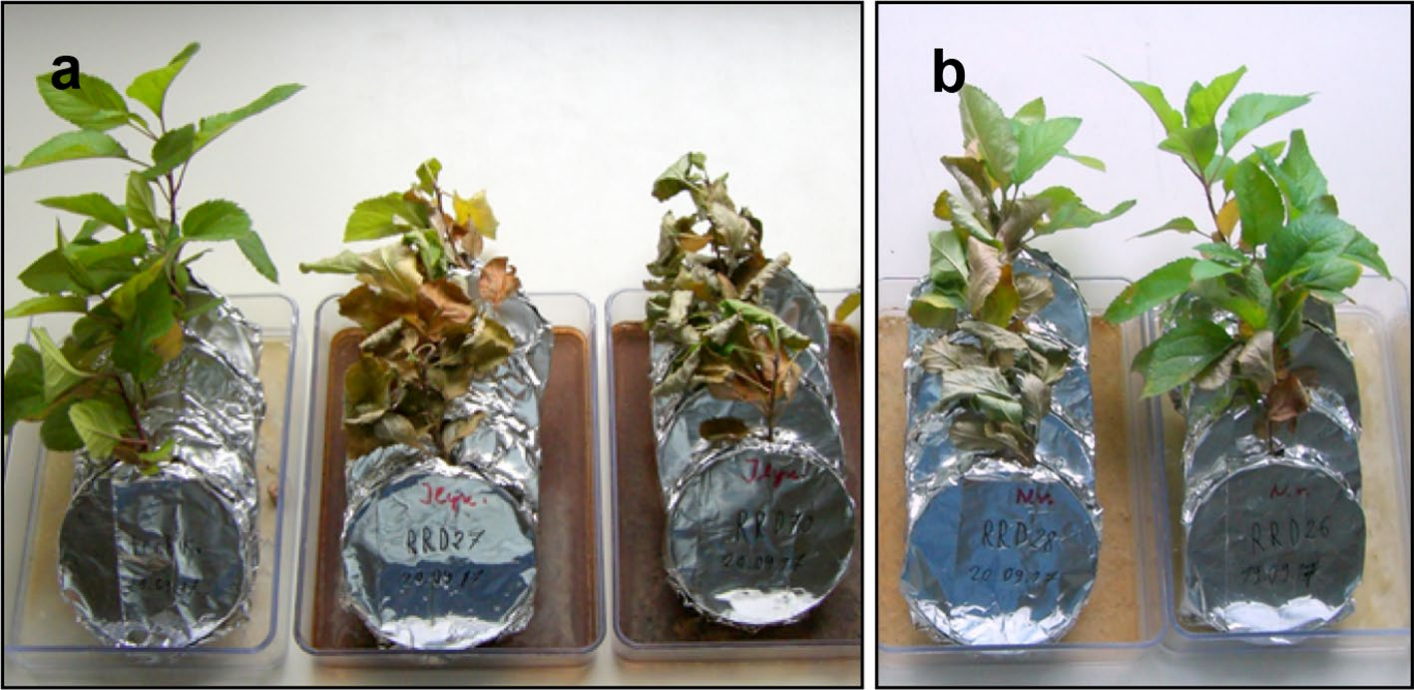
Perlite assay with M26 test plants grown in pure Perlite plus malt extract, plant reactions after 14 days. Non-inoculated control, left, and plants inoculated with isolates RRD 27 and RRD 70, both *Ilyonectria robusta* obtained from *R. corymbifera* in RRD soil from the site Heidgraben (**a**). Plants inoculated with isolates RRD 28 and RRD 26 from the site Heidgraben, both identified as *Rugonectria rugulosa* by HIS sequencing (**b**).

The evaluation of the shoot symptoms 4 and 14 days, respectively, after the beginning of the test resulted in a yellowing of the leaves or a strong wilting, depending on the fungal isolate used. The two *I. robusta* isolates caused the most severe wilting which started after only four days and soon led to the death of the plants (Fig. 10a). Leaf symptoms in plants inoculated with *R. rugulosa* isolates ranged from light yellowing to wilting of the lower leaves. Isolate RRD 26 showed slighter symptoms than isolate RRD 28. However, the plants survived over the entire test period of seven weeks (Fig. 10b). The non-inoculated M26 plants (controls) remained healthy and green throughout the test. Assessing the root symptoms macroscopically, severe necroses and browning of the total root systems were found in plants infected by *I. robusta* (Fig. ESM 10). In contrast, the roots of the plants infected with *R. rugulosa* continued to function in part and formed new secondary roots. This was reflected in the respective shoot symptoms.

In microscopic analyses, the cell and tissue reactions in fine root samples were studied over a period of seven weeks in order to attribute individual symptoms to the isolates tested. Only *R. rugulosa* isolates induced intracellular CF structures and cell necroses, as was detected before in fine root tissue of *R. corymbifera*. Cytoplasmic inclusion bodies were only induced by *I*. *robusta* and were occasionally present in cell groups with fungal infections. Brown chlamydospores occurred often inside and outside of the root (Fig. ESM 11a). Later on, the symptom of blackening started in single cells and extended over several neighbouring cells (Fig. ESM 11b,c). The host cells of M26 reacted with the formation of callose to prevent the fungus from entering, but failed as indicated by tissue browning, necrotic host cell collapse, and the development of intracellular hyphae and CF structures. The results obtained from eight plants per treatment are summarised in Table 2.

The Perlite assay showed that *R. rugulosa* isolates were responsible for the formation of CF structures in the outer tissue layers of apple roots. They had the ability to colonise the root tissue intercellularly after penetrating the root and to cause necrotic plant cell reactions, which led, in part, to the symptom of blackening. In contrast, the two *I. robusta* isolates caused neither CF structures nor blackening during apple root infections in this test. Here, black-brown cytoplasmic inclusions were found in the areas of intracellular fungal hyphae. Further, chlamydospores of *I. robusta* were detectable outside and inside of fine roots, often in large numbers.

## Discussion

The replant disease of roses is not a new phenomenon, but has been studied in less detail than ARD. One of our aims was to apply the diagnostic parameters developed for ARD to investigate the development of RRD symptoms in rose roots and to look for similarities in RRD and ARD. This should broaden the aspect of the causes of these economically important diseases. As long as the main factors and in particular the most important microbial actors are not yet defined, it is urgently necessary to identify early symptoms and to intensify the determination of their inducers. Therefore, we have characterized symptoms in fine roots of *R. corymbifera* ‘Laxa’, a commonly used and replant disease susceptible rootstock, in a greenhouse bio-test with soils affected by RRD and with techniques established in ARD tests^28,37,38^. Baumann et al.^37^ have shown with growth data of *R. corymbifera* 'Laxa' that the severity of the RRD was significantly more pronounced in soil from the Heidgraben site than from the Sangerhausen site. This difference was partly reflected in our fine root analyses. Our studies show that the diagnostic parameters developed for the early detection of ARD^28^ can support an early evidence of RRD in rose roots in replant affected soils. Several parameters like hair root status, structural damage, cell damages and especially the frequencies of microorganisms in affected fine root tissues can serve as first signs of the disease in rose. The leading cell damage symptoms of RD in apple roots, namely necrosis, blackening and cytoplasmic inclusions, were present in diseased fine roots of rose, but could not be statistically proven as significant characteristics of RRD, neither at the early investigation time point after three weeks in experiment 2 (Fig. ESM 8b) nor after nine weeks (Fig. ESM 4). Since 30 root segments per plant were analysed, the precision of the estimated proportions per plant is relatively high. But, as the microscopic evaluation of fresh material is very labour-intensive with such a large number of root segments and different disease parameters, only a limited number of plants (four or six) were examined in the experiments. Therefore, the estimation of average proportions based on the few single plants is relatively imprecise, as are their confidence intervals and comparisons. To improve the precision of significance tests at plant level in further experiments, it might be better to use more plants (e.g. 10–15) but fewer root segments per plant (e.g. 15–20), resulting in the same amount of labour. In addition to the analysis of symptom frequencies, the evaluation of symptom intensities could deepen and strengthen the results of early cell damage. Early diagnosis of root damage in a short-term bio-test contributes to the rapid detection of RRD, but cannot replace long-term growth tests for the assessment of replant disease-caused yield loss. Yao et al.^55^ and Polverigiani et al.^56^ reported this for ARD in intensive studies on apple root biomass, root morphology and architecture. Even more obvious than anatomical and morphological symptoms and often associated to these was the more pronounced colonisation of roots in both RRD soils with microorganisms, especially actinobacteria and fungi.

The second aim of the study was to assign characteristic symptoms to root pathogens that were repeatedly suspected of being causal factors of RRD but have never been correctly identified. Up to now, there have been no descriptions of fungal pathogens in root tissue of replant diseased rose plants. In particular, we were interested in the effects of pathogenic Nectriaceae because of frequently evaluated fungal mycelium and chlamydospores in necrotic infection sites in fine roots. Especially, the infection process and formation of CF structures are demonstrated for the first time in rose roots. The high proportion of endophytic Nectriaceae isolates in rose experiment 1 corroborates these observations and matches the results of a survey of fungal root endophytes obtained from replant diseased apple plants^36^. Interestingly, Popp et al. (2020) have also described and identified several genera and species of Nectriaceae in detail, which form CF structures in necrotic root cortex cells of replant-diseased apple rootstocks^57^. These findings support the hypothesis of a causal relation of these fungi to the induction of replant disease, at least in our tested soils. Moreover, based on long-term and in-depth research on Rosaceous small fruits in Northern Germany Weber and Entrop^17^ also suggested clarifying the significance of Nectriaceae in replant disease. This fungal group includes the *Cylindrocarpon*-like fungi, which were described as associated microorganisms in replant diseased apple and peach^13,32,35,58^. In addition, the high proportion of Nectriaceous fungi isolated out of tiny little infection sites in tissue samples of *R. corymbifera* ‘Laxa’ and the strong association of mycelium and the many CF structures in damaged cells of cortex parts—documented in whole mounts and thin sections—are in favour of our hypothesis: Nectriaceae fungi can induce the necrotic reactions and contribute to RRD. The results of the Perlite assay with *R. rugulosa* isolates from rose plants support this assumption. Future studies should include the set-up of an inoculation assay with rose plants and re-isolation of the inoculated fungi from rose roots. Moreover, observations including more plants and RRD soils from further sites are needed.

Besides the detailed microscopic assessment of fungal players in the causal complex of RRD, it was an essential step to check isolates from the infection sites for their identity. The first test of many isolates with ITS gene sequencing was obviously not specific enough to identify species and was therefore complemented with a multi-locus analysis to further characterise selected Nectriaceae isolates^59^. It turned out that the identification of Nectriaceae is subject to taxonomic advances and entails a constant evolution in the naming. In consequence, the results of the multi-locus analysis are ambiguous for some of our isolates, but should be seen as a growing insight into the identification of Nectriaceae. Gams and Jaklitsch^60^ critically addressed the problem of identifying and renaming of species on the basis of molecular analyses and gene bank deposits; this helps to explain our findings. The current detailed knowledge is due to the taxonomists who contributed significantly to the discovery of the diversity within the Nectriaceae in recent years^43,59,61–68^. It was beyond our goals and capacities to distinguish the isolates with an extended multi-locus analysis in combination with morphological features. With the four sets of primers used, we have achieved our goal of showing the spectrum of different species involved in the development of RRD symptoms, even in a single plant. Similar to our studies, Lawrence et al.^67^ identified, in a multi-locus sequence typing of four loci (ITS, TUB2, TEF1, and HIS), 12 *Cylindrocarpon*-like fungal species across five genera associated with black foot disease of grapevine and other diverse root diseases of fruit and nut crops in California. They stated that the HIS marker was essential, either singly or in combination with the other mentioned genes, for the precise identification of most *Dactylonectria* species. Our results in Supplement Table 2 confirm this observation.

In our tests, the RRD isolates of *R. rugulosa* (syn. *Neonectria rugulosa*, syn. *Nectria rugulosa*^62^) were unequivocally verified as root pathogens of rose. *Dactylonectria* spp. and *Ilyonectria* spp. were also determined to be main causative pathogens of Rosaceous small fruit root diseases^17^ and of the decline and root rot of loquat (*Eriobotrya japonica* ‘Algerie’)^15^. Furthermore, *Cylindrocarpon*-like species have been described in connection with root rot and decline effects in kiwifruit (*Actinidia chienensis*), avocado (*Persea americana*), olive (*Olea europaea*), walnut (*Juglans regia*), and grapevine (*Vitis vinifera*)^67^. This underlines the wide host spectrum of these pathogenic Nectriaceae, which goes beyond the Rosaceae. However, deviations between the aggressiveness of isolates may occur as was studied in the Perlite assay and has been shown in tests with *Dactylonectria torresensis* from apple roots^36^ and also for other *Cylindrocarpon*-like species in apple^31^ and *Vitis vinifera*^69^. Nevertheless, it should be taken into account in which test system the analysis is carried out, in artificial inoculation systems or natural soil tests. The detailed evaluation of Nectriaceae as a major cause of replant diseases, their attraction by plant root exudates as well as their interactions with other organisms of the disease complex will be extremely important challenges for future research.

By means of the artificial inoculations in the Perlite assay, it was established that similar fungal root endophytes induce the same necrotic plant reactions in cortical tissue of both rose and apple. In addition, the single infection tests on M26 in the Perlite assay supplied the evidence that some RRD isolates can actually form the CF structures, others cause cellular inclusion bodies and some lead to symptoms of necrosis and blackening. Therefore, there are some indications that in *Rosa* and *Malus* the same pathogens are involved in replant disease. This would also answer the question raised by Szabo^24^ of whether the pathogens in apple and rose are identical when RD occurs. This raises the important question of the specificity of replant diseases.

It remains less clear which role other microorganisms detected in rose fine roots play for the induction and development of RRD. There was no evidence for actinobacteria or other bacteria to damage cells as first pathogens; however, they frequently colonised the root surface and infected root tissue areas as simultaneous or subsequent invaders. This also applies to the yet unknown fungus with conspicuous arthroconidia formation on the surface of rose fine roots. The single celled, melanised conidia released from conidiogenous hyphae roughly resemble the conidia of *Leohumicola* spp.^70,71^, but isolations with standard media and identification tests failed so far.

Since rose root infections with Pythiaceae have been found very rarely and if so, in plants cultivated in untreated as well as in irradiated soil, these microbial actors were not further investigated, even though they had been reported as potential causes of ARD^27,33,72,73^. Pythiaceae should be tested in future inoculation tests to clarify their role in the rose replant disease complex. From our experiments, the identification of *Phytopythium vexans* as an intermediate of *Pythium* sp. to *Phytophthora* species^74^ presents new information in this context.

*Fusarium oxysporum* was sometimes isolated from necrotic infection sites in rose roots. This result confirms other reports on *Fusarium* species in the biotic complex of causal factors of ARD^34,75^. It cannot be ruled out from our studies, that *Fusarium* species may act in addition to Nectriaceae. In a recently performed Perlite assay, a *Fusarium oxysporum* isolate from apple plants cultured in ARD soil from the Heidgraben site caused wilting, but had no such impairing effects (cellular necrosis, blackening, and cytoplasmic inclusions) in root tissue that were observed after inoculation with *R. rugulosa*^36^. *Fusarium* spp. are considered to be minor players in causing ARD^30,76^, although they were reported as the most frequent root-colonising fungi^34^.

It was possible to detect mycorrhizal fungi in rose fine roots, known as potential beneficial symbionts. However, in our tests with *R. corymbifera* 'Laxa' histological studies showed that the mycorrhizal fungi present at site Heidgraben did not protect against severe infections and cell damage caused by Nectriaceae. In undisturbed soils, the autochthonous mycorrhiza contributes to maintaining the health of plants^77^. Thus, the benefits of symbiotic arbuscular mycorrhizal fungi were investigated and discussed as a remedy in ARD^78^. Čatská^79^ and Mehta and Bharat^80^ reported promising effects on the productivity of apple plants in ARD soils after inoculation with *Glomus fasciculatum* isolates. In a pot tests with peach, *Acaulospora scrobiculata* achieved mitigation of RD due to modulating the soil microbe balance, improving soil aggregate stability, and changing the root exudate compositions^21^. In our experiment 1 the biocontrol capacity of the autochthonous mycorrhiza on root pathogens in *R. corymbifera* ‘Laxa’ seemed to be limited, at least in the tested early growth phase.

In conclusion, this study provided evidence that, (a) early symptoms of replant diseased fine roots can be similar in apple and rose and can help identify the disease within a few weeks of testing, (b) fungal root endophytes can induce the development of RD symptoms, (c) histological investigations confirmed the symptomatic plant cell reactions on fungal infections by members of the Nectriaceae, and (d) a variety of pathogenic genera and species of the Nectriaceae are worthwhile to be studied in detail regarding their contribution to the RRD complex. However, it is difficult to generalise our findings, obtained with fungi isolated from a limited number of rose plants grown in only two RRD soils. This calls for a wider study in which RRD fungi are isolated from a large number of plants collected at several different sites. We would recommend performing the short-term biotest with a duration of 3–8 weeks, if fast results are required for the determination of RD.

## Supplementary Information

Supplementary Figure 1.

Supplementary Figure 2.

Supplementary Figure 3.

Supplementary Figure 4.

Supplementary Figure 5.

Supplementary Figure 6.

Supplementary Figure 7.

Supplementary Figure 8.

Supplementary Figure 9.

Supplementary Figure 10.

Supplementary Figure 11.

Supplementary Table 1.

Supplementary Table 2.
