# Author Correction: Observations on early fungal infections with relevance for replant disease in fine roots of the rose rootstock *Rosa corymbifera* 'Laxa'

**DOI:** 10.1038/s41598-021-95182-5

**Published:** 2021-08-19

**Authors:** G. Grunewaldt‑Stöcker, C. Popp, A. Baumann, S. Fricke, M. Menssen, T. Winkelmann, E. Maiss

**Affiliations:** 1 Institute of Horticultural Production Systems, Section Phytomedicine, Leibniz Universität Hannover, Herrenhäuser Str. 2, 30419 Hannover, Germany; 2 Institute of Horticultural Production Systems, Section of Woody Plant and Propagation Physiology, Leibniz Universität Hannover, Herrenhäuser Str. 2, 30419 Hannover, Germany; 3 Department of Biostatistics, Institute of Cell Biology and Biophysics, Leibniz Universität Hannover, Herrenhäuser Str. 2, 30419 Hannover, Germany

Correction to: *Scientific Reports* https://doi.org/10.1038/s41598-020-79878-8, published online 29 December 2020

The Supplementary Table 2 file published with this Article was incomplete.

The Gene Bank Accessions numbers for genes Histone 3 (HIS), partial β-tubulin (TUB) and translation elongation factor 1-α (TEF) were omitted.

The original Supplementary Table 2 file is provided below.

This error has now been corrected in the Supplementary Information file that accompanies the original Article.

## Supplementary Information

Supplementary Table 2.

