# Supplementary material for: Observations on early fungal infections with relevance for replant disease in fine roots of the rose rootstock Rosa corymbifera 'Laxa'
Source: Sci Rep. 2020 Dec 29;10:22410. doi: 10.1038/s41598-020-79878-8 (PMC7772344; doi:10.1038/s41598-020-79878-8)
Supplement: Supplementary file 1 — Supplementary Figure 1. [file 41598_2020_79878_MOESM1_ESM.docx]

**Observations on early fungal infections with relevance for replant disease in fine roots of the rose rootstock *Rosa corymbifera* 'Laxa'**

by G. Grunewaldt-Stöcker, C. Popp, A. Baumann, S. Fricke, M. Menssen, T. Winkelmann, E. Maiss.


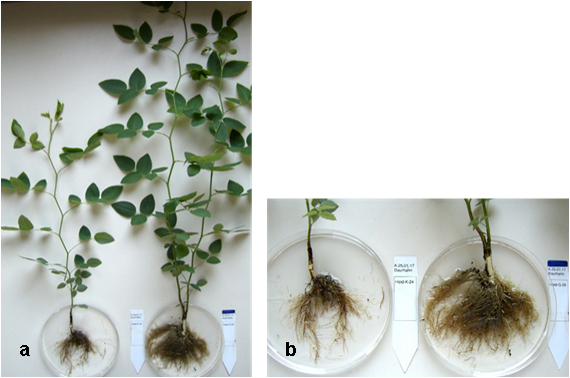


**Fig. ESM 1** *R. corymbifera* ‘Laxa’ after 9 weeks of cultivation in untreated (left) and irradiated (right) RRD soil from the site Heidgraben (a), and close-up view of root systems (b)
