# Supplementary material for: Observations on early fungal infections with relevance for replant disease in fine roots of the rose rootstock Rosa corymbifera 'Laxa'
Source: Sci Rep. 2020 Dec 29;10:22410. doi: 10.1038/s41598-020-79878-8 (PMC7772344; doi:10.1038/s41598-020-79878-8)
Supplement: Supplementary file 2 — Supplementary Figure 2. [file 41598_2020_79878_MOESM2_ESM.docx]

**Observations on early fungal infections with relevance for replant disease in fine roots of the rose rootstock *Rosa corymbifera* 'Laxa'**

by G. Grunewaldt-Stöcker, C. Popp, A. Baumann, S. Fricke, M. Menssen, T. Winkelmann, E. Maiss.


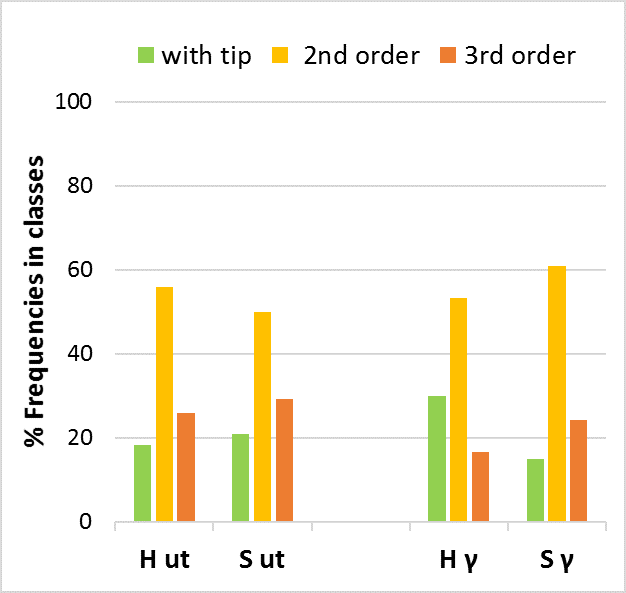


**Fig. ESM 2** Distribution of analysed fine root segments of *R. corymbifera* ‘Laxa’ according to their position in the root system. % Frequencies of segments in three classes were assessed after 9 weeks of cultivation in untreated (ut) and irradiated (γ) soils from replant diseased sites Heidgraben (H) and Sangerhausen (S), respectively. n=120 root segments pooled from four plants
