# Supplementary material for: Observations on early fungal infections with relevance for replant disease in fine roots of the rose rootstock Rosa corymbifera 'Laxa'
Source: Sci Rep. 2020 Dec 29;10:22410. doi: 10.1038/s41598-020-79878-8 (PMC7772344; doi:10.1038/s41598-020-79878-8)
Supplement: Supplementary file 3 — Supplementary Figure 3. [file 41598_2020_79878_MOESM3_ESM.docx]

**Observations on early fungal infections with relevance for replant disease in fine roots of the rose rootstock *Rosa corymbifera* 'Laxa'**

by G. Grunewaldt-Stöcker, C. Popp, A. Baumann, S. Fricke, M. Menssen, T. Winkelmann, E. Maiss.


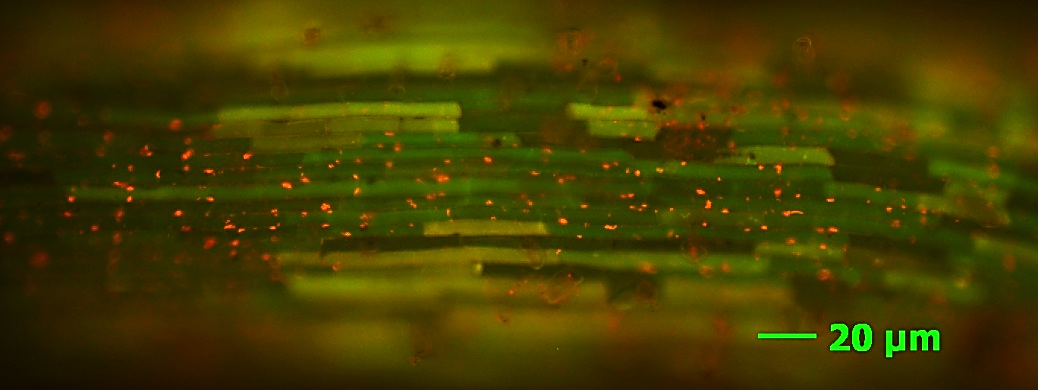


**b**


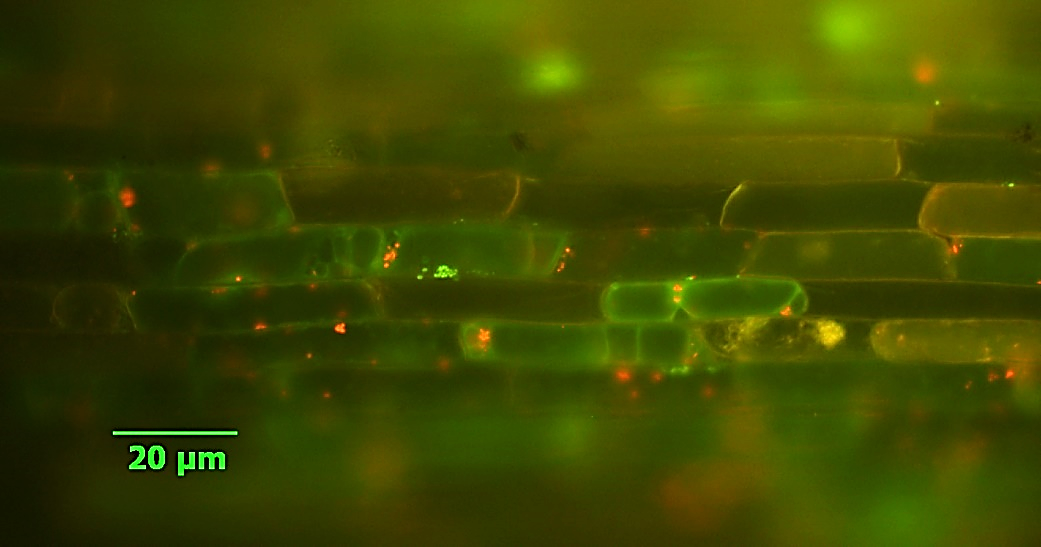


**a**


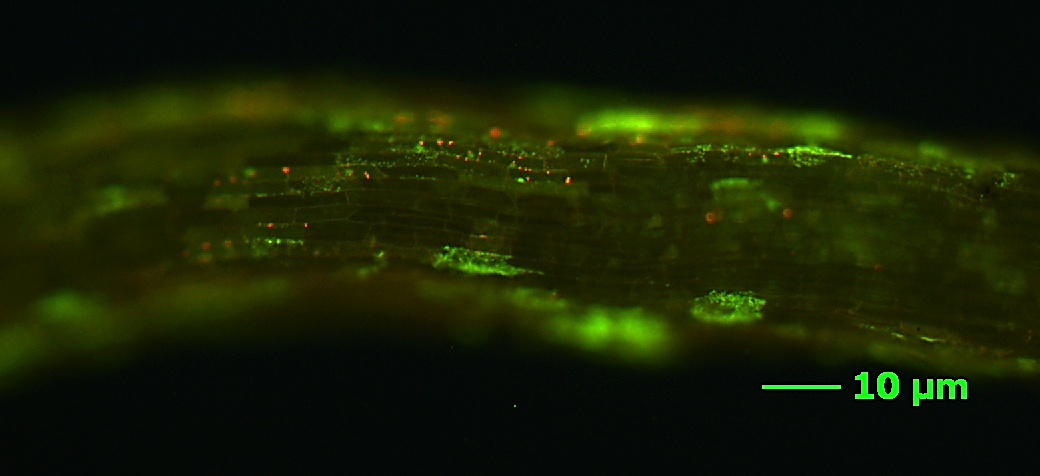


**c**

**Fig. ESM 3** Examples of vital cell staining in fine roots of *R. corymbifera* ‘Laxa’ with a close-up of greenish vital cells with red intravacuolar stain particles (a), with a band of vital cells between dead (yellow) and necrotic (black) cells (b), and with several vital cells in necrotic tissue, in focus with green fluorescent cells infected by Actinobacteria (arrows, c). Epifluorescence technique after FUN®1 cell staining
