# Supplementary material for: Observations on early fungal infections with relevance for replant disease in fine roots of the rose rootstock Rosa corymbifera 'Laxa'
Source: Sci Rep. 2020 Dec 29;10:22410. doi: 10.1038/s41598-020-79878-8 (PMC7772344; doi:10.1038/s41598-020-79878-8)
Supplement: Supplementary file 4 — Supplementary Figure 4. [file 41598_2020_79878_MOESM4_ESM.docx]

**Observations on early fungal infections with relevance for replant disease in fine roots of the rose rootstock *Rosa corymbifera* 'Laxa'**

by G. Grunewaldt-Stöcker, C. Popp, A. Baumann, S. Fricke, M. Menssen, T. Winkelmann, E. Maiss.


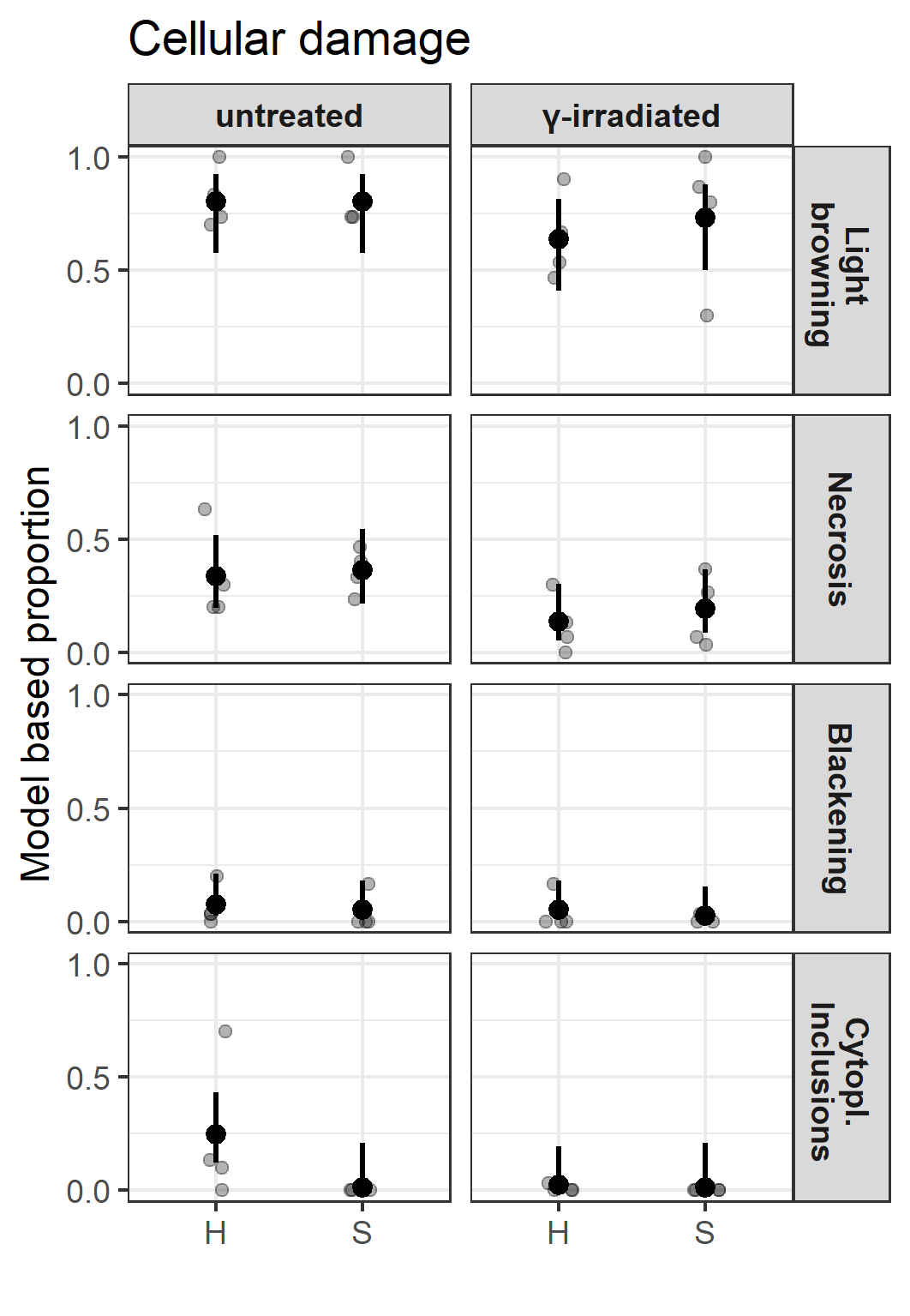

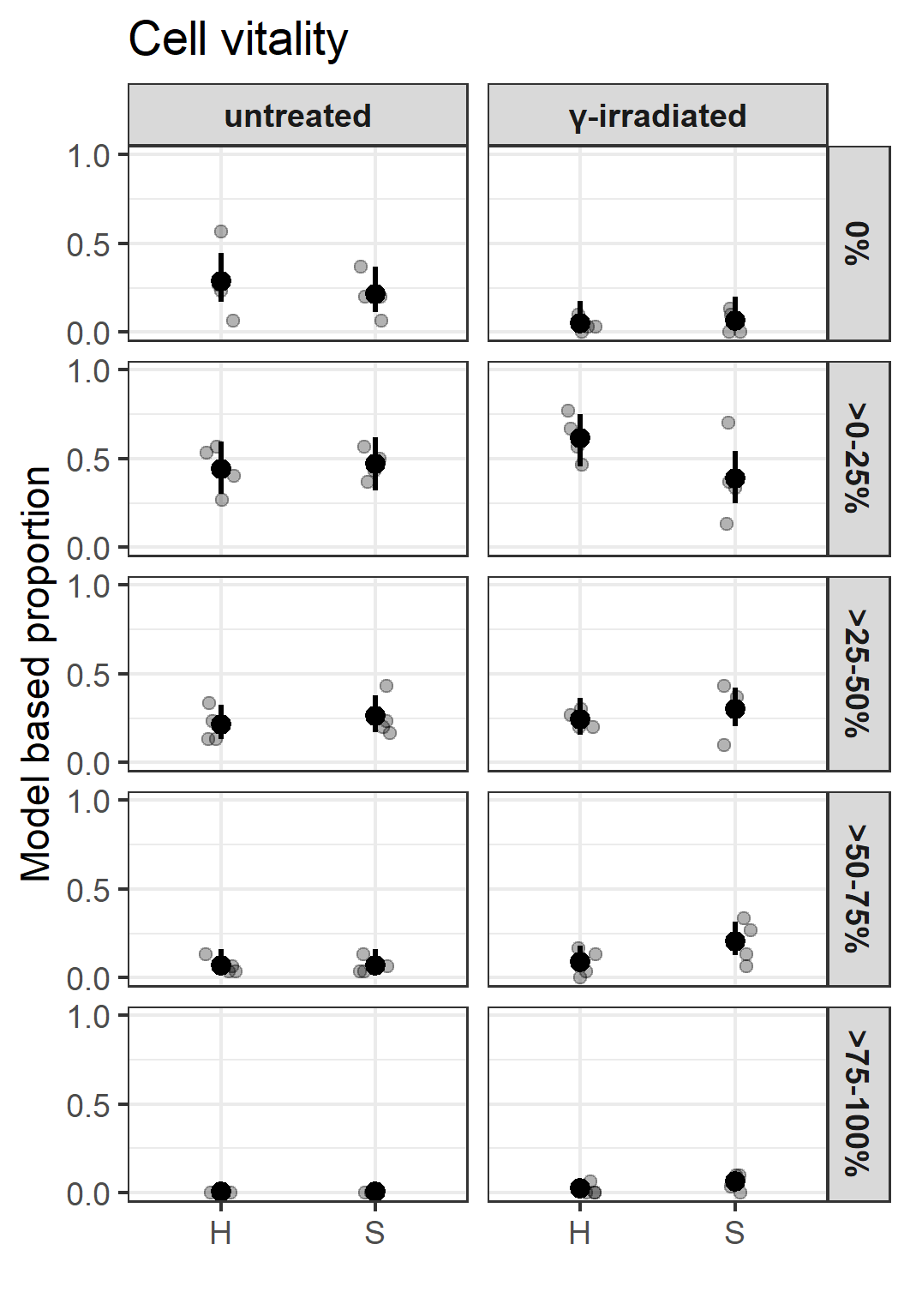


**a**

**b**

**ESM Fig. 4** Cellular damages and discoloration (a) and cell vitality in the outer cortex cell layers (b) in *R. corymbifera* ‘Laxa’ fine roots after 9 weeks of cultivation in untreated or γ-irradiated replant-diseased soils from Heidgraben (H) and Sangerhausen (S), respectively. Proportions of cellular damage for each treatment and four symptom classes, of cell vitality in segments rated in five classes from 0 to 100% after FUN®1 cell staining. Given are observed proportions per plant (grey dots), mean proportions driven from the models (black dots), and 95% confidence intervals for the mean proportions (black bars) from 30 root segments of n=4 plants. If α=0.05 no significant differences between mean proportions could be found. This figure was created using the software R version 3.6.1 (R Core Team 2019, [https://www.R-project.org/](https://www.r-project.org/))^48, 51, 52, 54^.
