# Supplementary material for: Observations on early fungal infections with relevance for replant disease in fine roots of the rose rootstock Rosa corymbifera 'Laxa'
Source: Sci Rep. 2020 Dec 29;10:22410. doi: 10.1038/s41598-020-79878-8 (PMC7772344; doi:10.1038/s41598-020-79878-8)
Supplement: Supplementary file 5 — Supplementary Figure 5. [file 41598_2020_79878_MOESM5_ESM.docx]

**Observations on early fungal infections with relevance for replant disease in fine roots of the rose rootstock *Rosa corymbifera* 'Laxa'**

by G. Grunewaldt-Stöcker, C. Popp, A. Baumann, S. Fricke, M. Menssen, T. Winkelmann, E. Maiss.


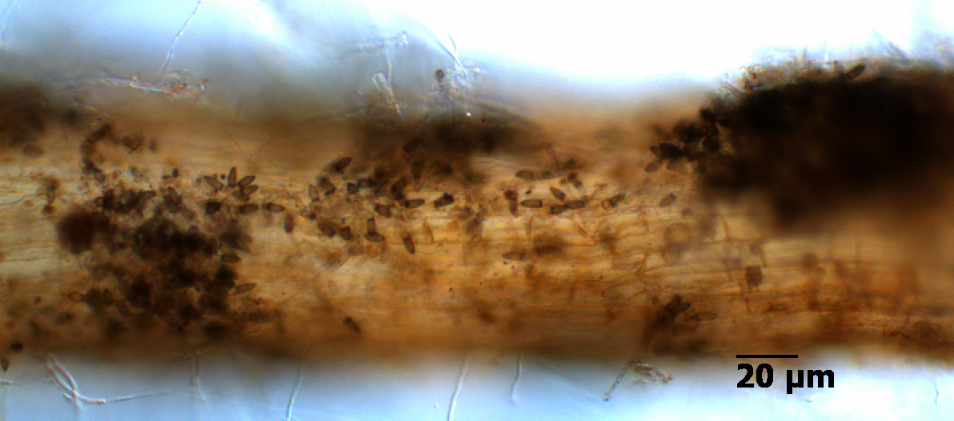


**a**


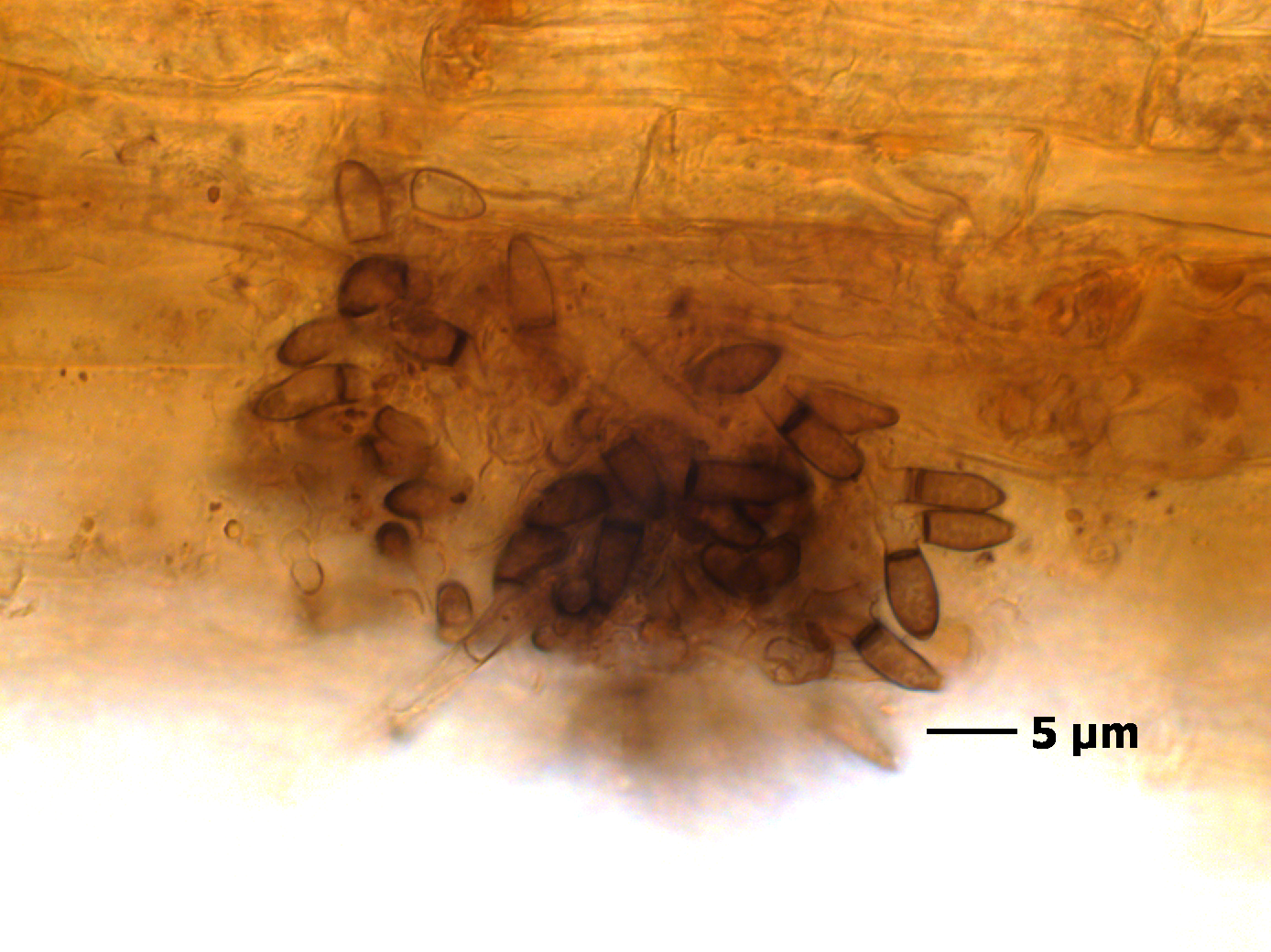


**b**

**Fig. ESM 5** Development of arthroconidia on the surface of *R. corymbifera* ‘Laxa’ roots by a yet unidentified fungal endophyte 9 weeks after cultivation in untreated RRD soil from site Heidgraben (a) and infection of rhizodermal cells (b), unstained root samples
