# Supplementary material for: Observations on early fungal infections with relevance for replant disease in fine roots of the rose rootstock Rosa corymbifera 'Laxa'
Source: Sci Rep. 2020 Dec 29;10:22410. doi: 10.1038/s41598-020-79878-8 (PMC7772344; doi:10.1038/s41598-020-79878-8)
Supplement: Supplementary file 7 — Supplementary Figure 7. [file 41598_2020_79878_MOESM7_ESM.docx]

**Observations on early fungal infections with relevance for replant disease in fine roots of the rose rootstock *Rosa corymbifera* 'Laxa'**

by G. Grunewaldt-Stöcker, C. Popp, A. Baumann, S. Fricke, M. Menssen, T. Winkelmann, E. Maiss.


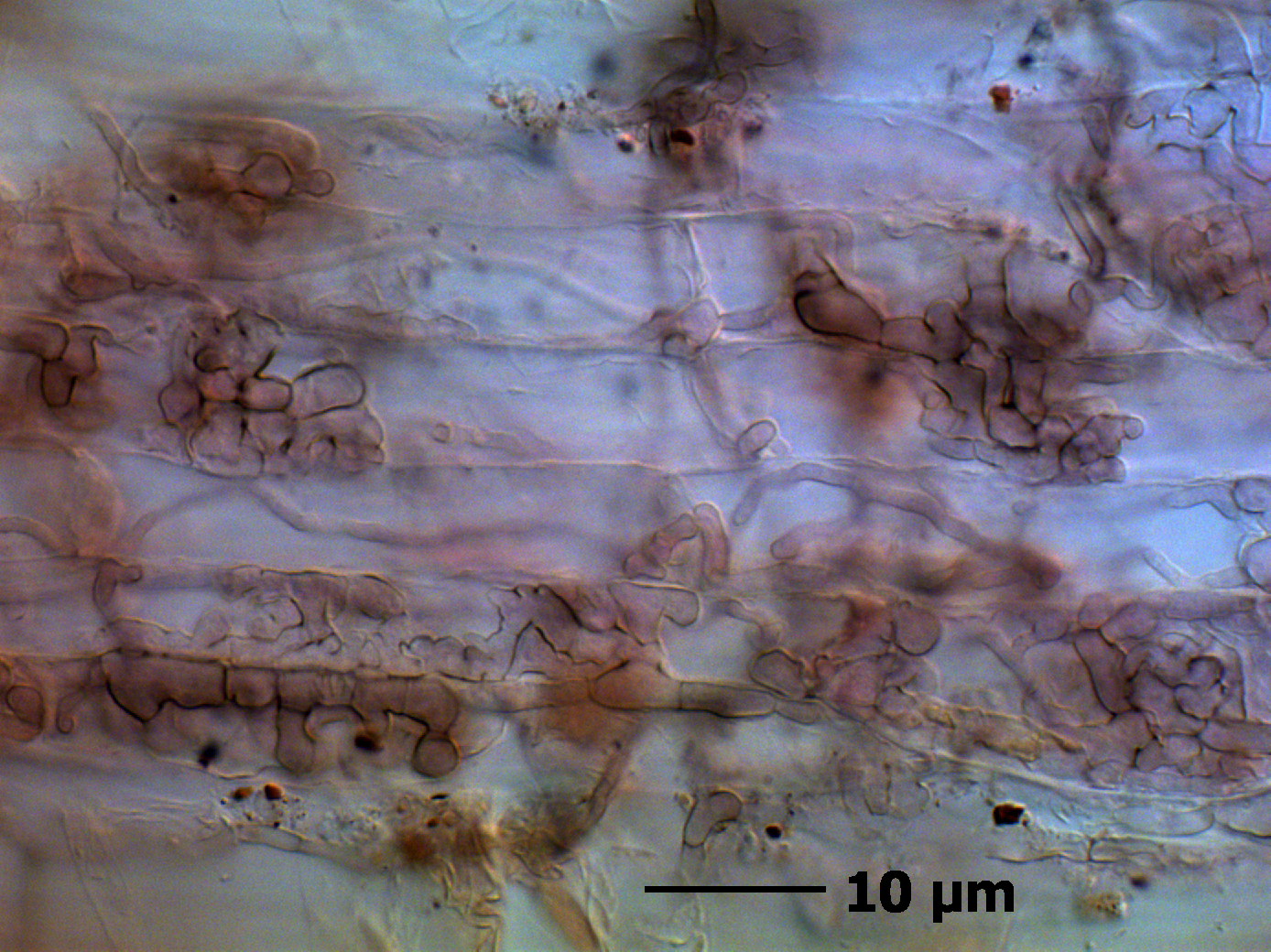


**Fig. ESM 7** Colonisation of *R. corymbifera* ‘Laxa’ fine root tissue by a dark septate endophyte (DSE) with melanised cell groups, after six weeks of cultivation in untreated RRD soil from site Heidgraben (H ut), FUN®1 cell stain, bright field microscopy
