# Supplementary material for: Observations on early fungal infections with relevance for replant disease in fine roots of the rose rootstock Rosa corymbifera 'Laxa'
Source: Sci Rep. 2020 Dec 29;10:22410. doi: 10.1038/s41598-020-79878-8 (PMC7772344; doi:10.1038/s41598-020-79878-8)
Supplement: Supplementary file 8 — Supplementary Figure 8. [file 41598_2020_79878_MOESM8_ESM.docx]

**Observations on early fungal infections with relevance for replant disease in fine roots of the rose rootstock *Rosa corymbifera* 'Laxa'**

by G. Grunewaldt-Stöcker, C. Popp, A. Baumann, S. Fricke, M. Menssen, T. Winkelmann, E. Maiss.


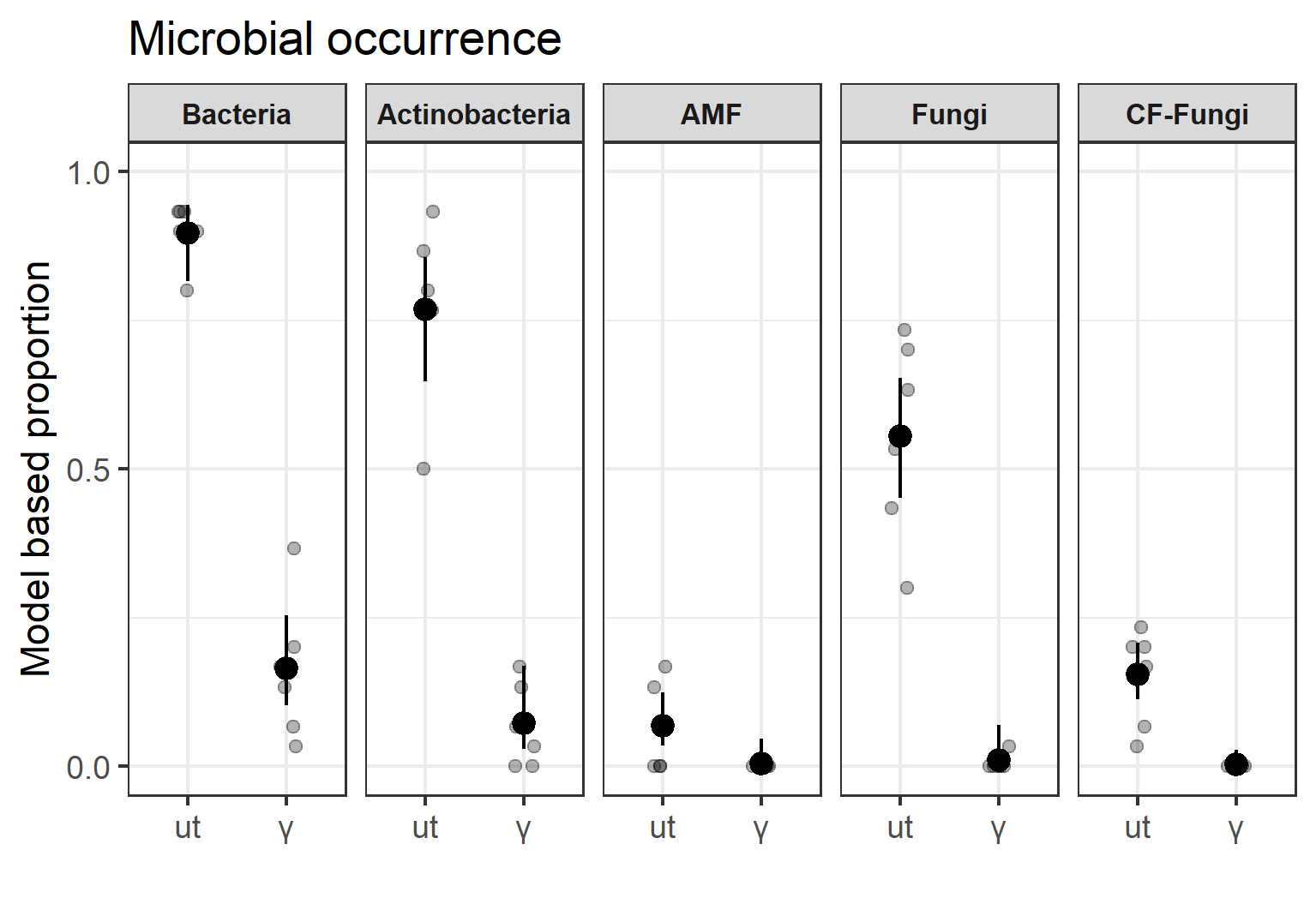

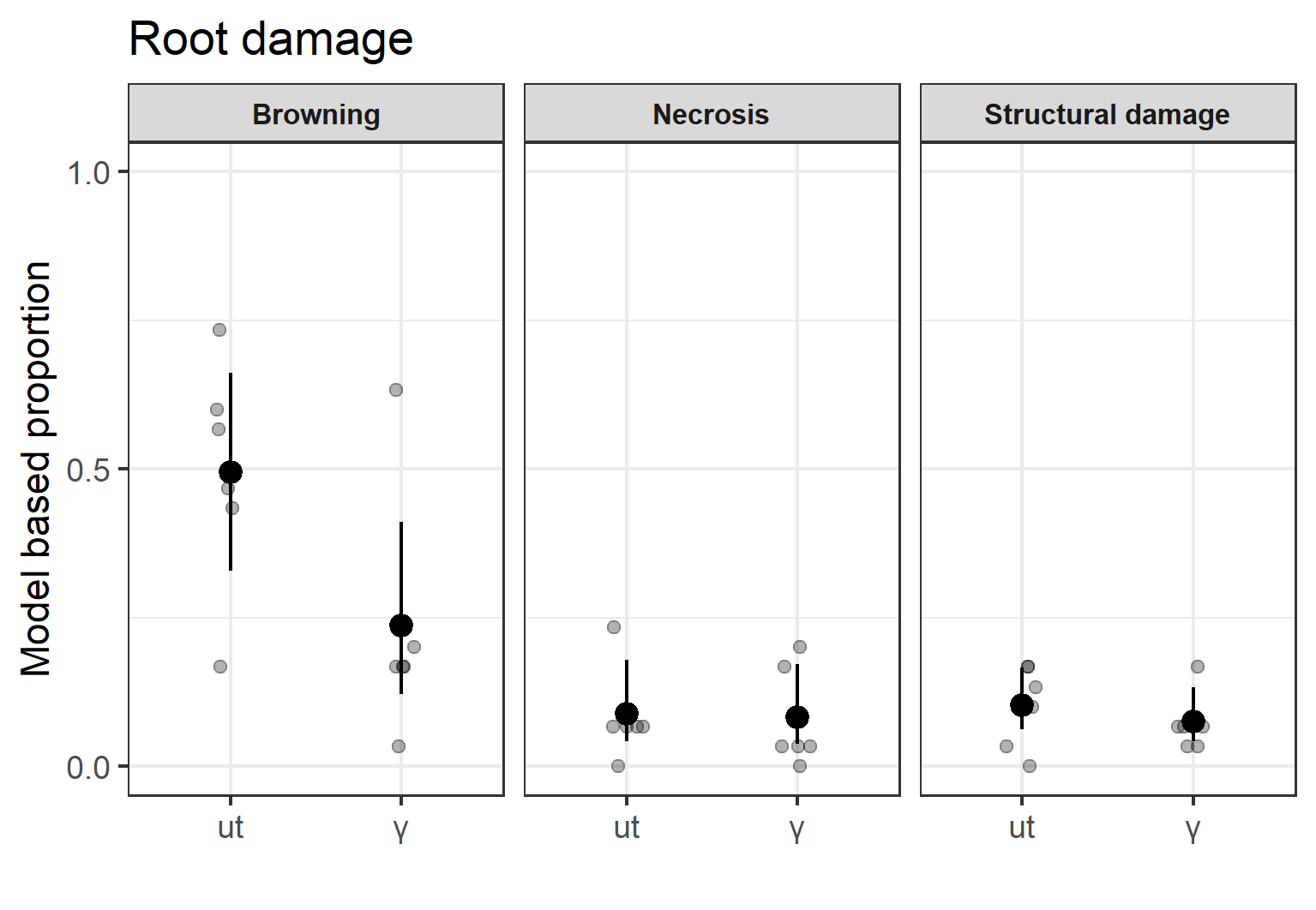


**b**

**c**


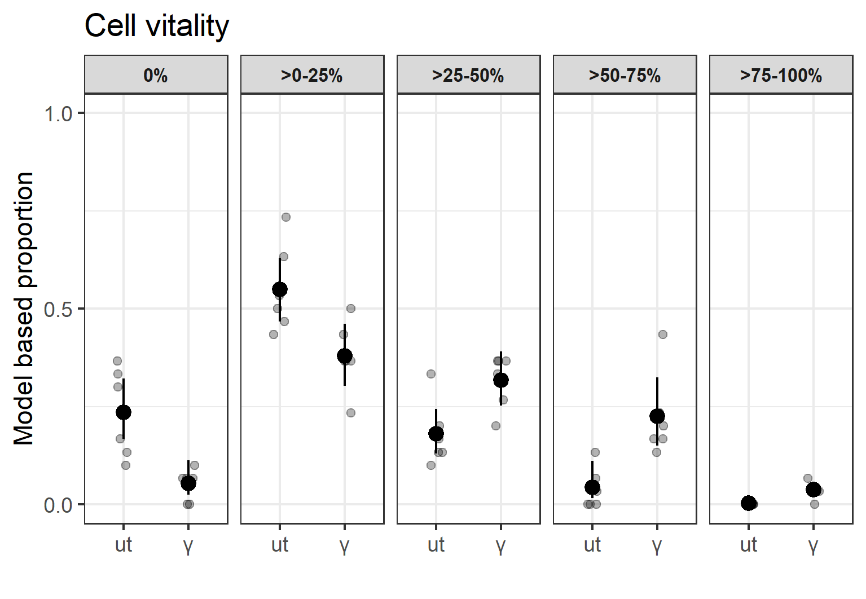


**a**

**Fig. ESM 8** Cell vitality (a), root damage (b), and microbial occurrence (c) in fine roots of *R. corymbifera* ‘Laxa’ grown for three weeks in replant-diseased Heidgraben soils, either untreated (ut) or γ-irradiated (γ) in experiment 2. Given are observed proportions per plant (grey dots), mean proportions driven from the models (black dots), and 95% confidence intervals for the mean proportions (black bars) from 30 root segments of n 6 plants. Significant differences (p=0.05) in a) for all classes of cell vitality (ut vs. γ); significant difference (p=0.1) in b) for browning (ut vs. γ); significant differences (p=0.05) in c) for Bacteria, Actinobacteria, Fungi and CF-fungi (ut vs. γ). This figure was created using the software R version 3.6.1 (R Core Team 2019, [https://www.R-project.org/](https://www.r-project.org/))^48, 51, 52, 54^.
