# Supplementary material for: Observations on early fungal infections with relevance for replant disease in fine roots of the rose rootstock Rosa corymbifera 'Laxa'
Source: Sci Rep. 2020 Dec 29;10:22410. doi: 10.1038/s41598-020-79878-8 (PMC7772344; doi:10.1038/s41598-020-79878-8)
Supplement: Supplementary file 9 — Supplementary Figure 9. [file 41598_2020_79878_MOESM9_ESM.docx]

**Observations on early fungal infections with relevance for replant disease in fine roots of the rose rootstock *Rosa corymbifera* 'Laxa'**

by G. Grunewaldt-Stöcker, C. Popp, A. Baumann, S. Fricke, M. Menssen, T. Winkelmann, E. Maiss.


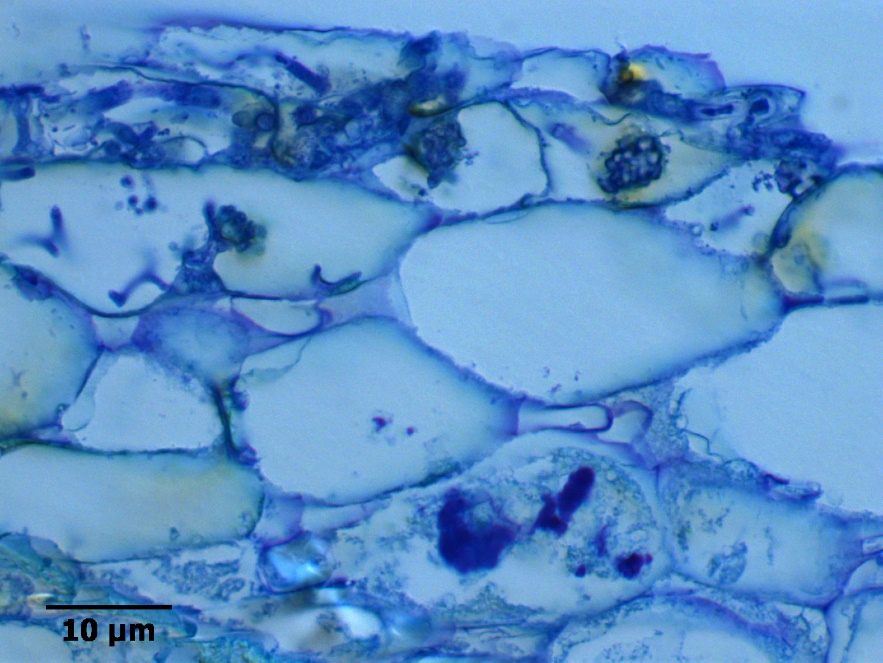

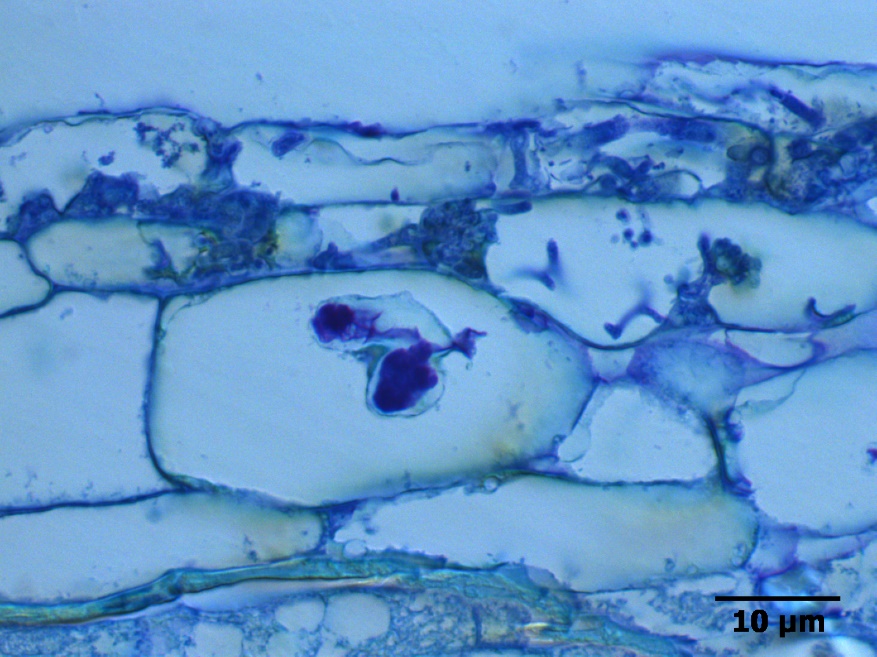


**a**


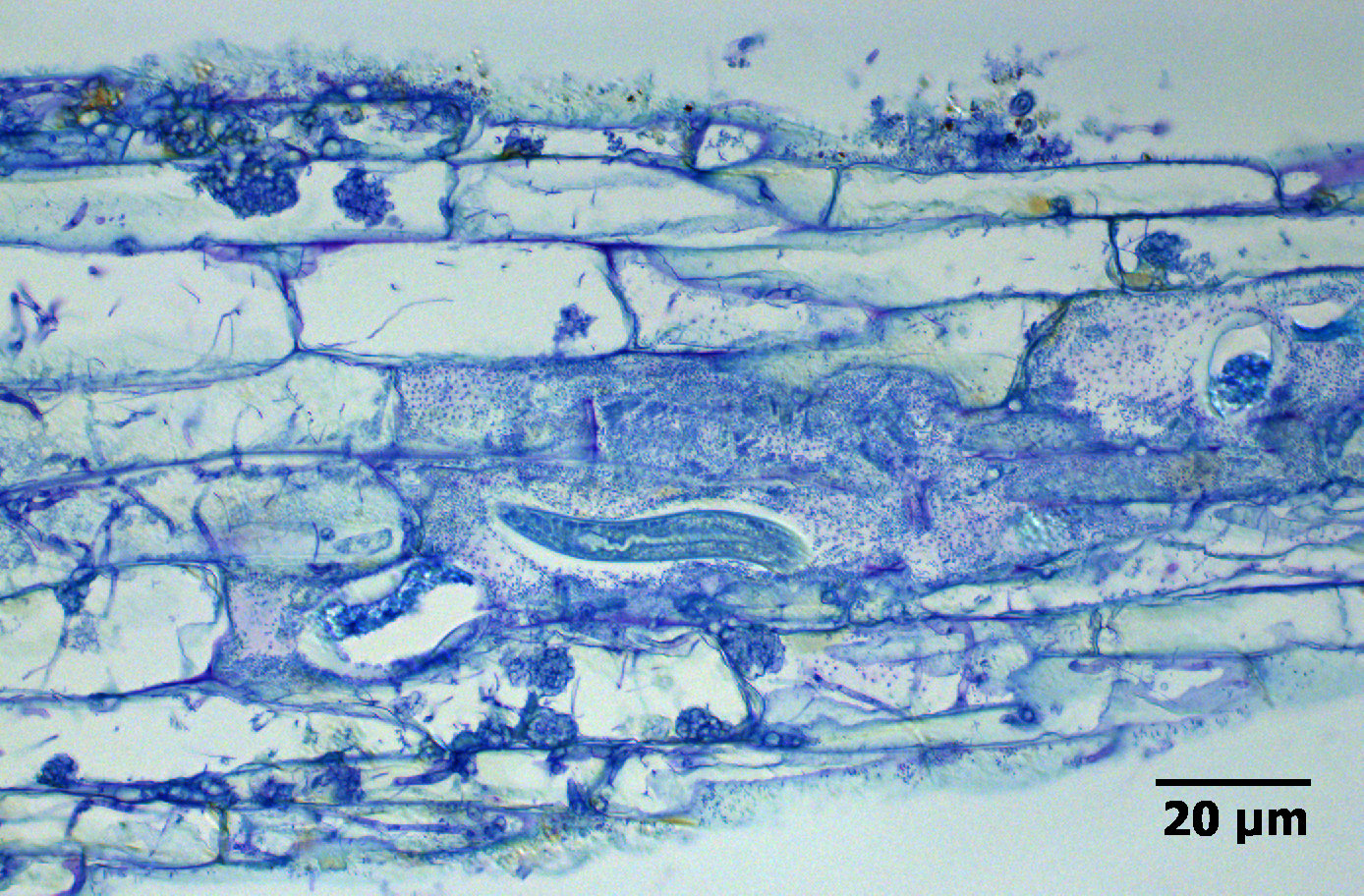


**b**

**Fig. ESM 9** Thin sections of *R. corymbifera* ‘Laxa’ fine roots, after 9 weeks of cultivation in untreated RRD soil from site Heidgraben. Infected cortex cells with degenerated mycorrhizal arbuscules (arrows) surrounded in the outer cell layers by cells with fungal CF structures (a), and cortex tissue with fungal infections and bacterial colonisation in a nematode-infested area with destroyed cell structures (b)
