# Supplementary material for: Observations on early fungal infections with relevance for replant disease in fine roots of the rose rootstock Rosa corymbifera 'Laxa'
Source: Sci Rep. 2020 Dec 29;10:22410. doi: 10.1038/s41598-020-79878-8 (PMC7772344; doi:10.1038/s41598-020-79878-8)
Supplement: Supplementary file 10 — Supplementary Figure 10. [file 41598_2020_79878_MOESM10_ESM.docx]

**Observations on early fungal infections with relevance for replant disease in fine roots of the rose rootstock *Rosa corymbifera* 'Laxa'**

by G. Grunewaldt-Stöcker, C. Popp, A. Baumann, S. Fricke, M. Menssen, T. Winkelmann, E. Maiss.


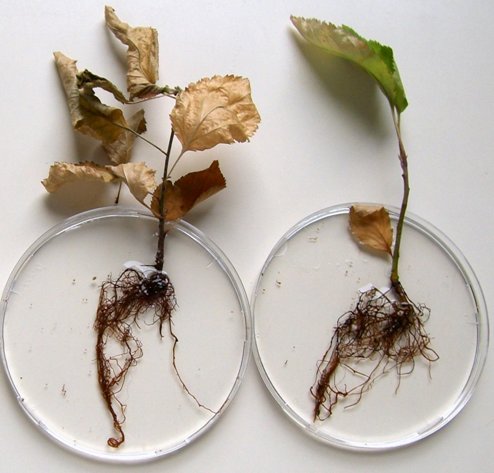


**Fig. ESM 10** Totally necrotic roots of dead M26 apple plantlets in the Perlite assay 38 days after inoculation with *Ilyonectria robusta* isolate RRD 70 (left), and *Ilyonectria robusta* isolate RRD 27 (right)
