# Supplementary material for: Observations on early fungal infections with relevance for replant disease in fine roots of the rose rootstock Rosa corymbifera 'Laxa'
Source: Sci Rep. 2020 Dec 29;10:22410. doi: 10.1038/s41598-020-79878-8 (PMC7772344; doi:10.1038/s41598-020-79878-8)
Supplement: Supplementary file 11 — Supplementary Figure 11. [file 41598_2020_79878_MOESM11_ESM.docx]

**Observations on early fungal infections with relevance for replant disease in fine roots of the rose rootstock *Rosa corymbifera* 'Laxa'**

by G. Grunewaldt-Stöcker, C. Popp, A. Baumann, S. Fricke, M. Menssen, T. Winkelmann, E. Maiss.


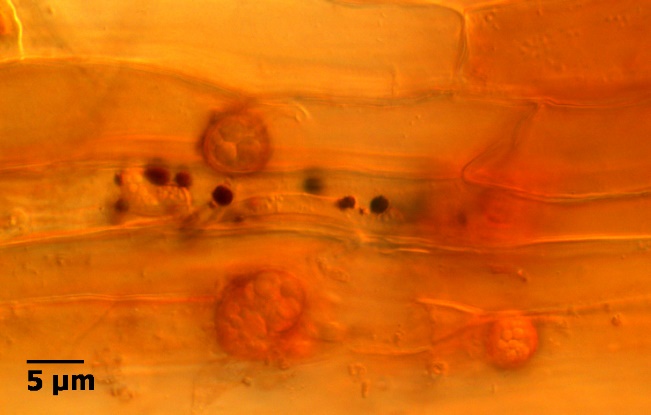


**a**


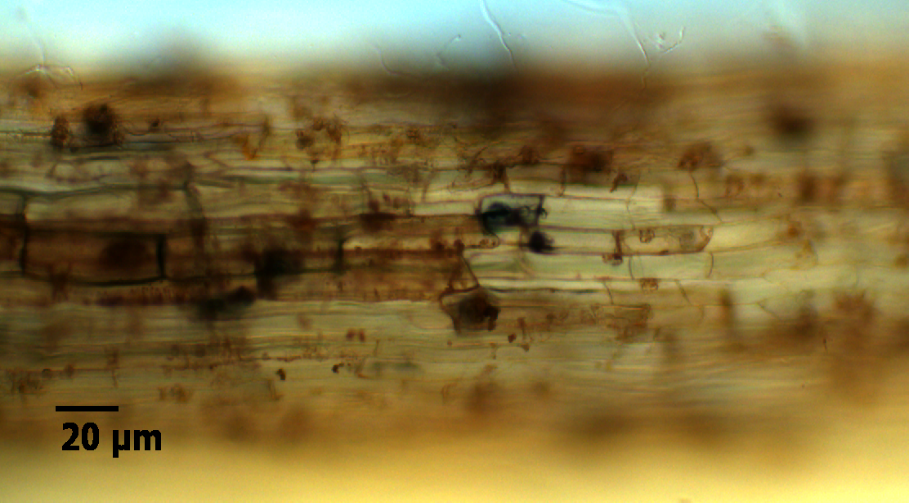


**b**


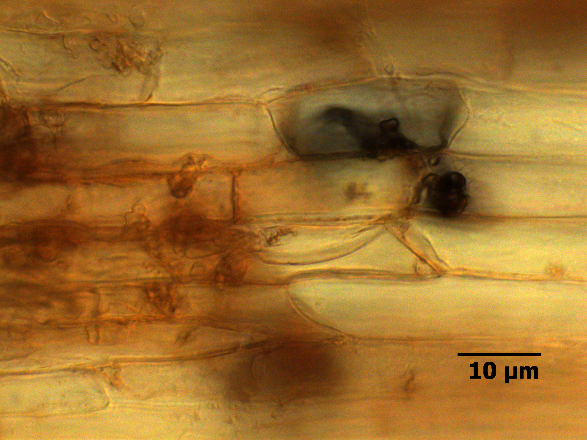


**c**

**Fig. ESM 11** Fine roots of M26 apple plantlets in the Perlite assay after inoculation with *Ilyonectria robusta* isolate RRD 70 and *Rugonectria rugulosa* isolate RRD 28. Brown chlamydospores and black intracellular inclusion bodies with isolate RRD 70, 14 dpi (a). The symptom of blackening in single cells with RRD 28, 51 dpi (b, c)
