# Supplementary material for: Observations on early fungal infections with relevance for replant disease in fine roots of the rose rootstock Rosa corymbifera 'Laxa'
Source: Sci Rep. 2020 Dec 29;10:22410. doi: 10.1038/s41598-020-79878-8 (PMC7772344; doi:10.1038/s41598-020-79878-8)
Supplement: Supplementary file 12 — Supplementary Table 1. [file 41598_2020_79878_MOESM12_ESM.docx]

**Observations on early fungal infections with relevance for replant disease fine roots of the rose rootstock *Rosa corymbifera* 'Laxa'**

by G. Grunewaldt-Stöcker, C. Popp, A. Baumann, S. Fricke, M. Menssen, T. Winkelmann, E. Maiss.

**Table ESM 1** Identification of fungal root endophytes in experiment 1: 49 isolates (F) from the first screening of surface sterilized root samples and 84 isolates (RRD) from selected infection sites that were gained from *Rosa corymbifera* ‘Laxa’ plants after cultivation in RRD soil. Sanger sequencing of internal transcribed spacer (**ITS**) region and Accession Numbers in the Genebank

| **Isolate Designation** | **Identified as** | **Accession Nr.** |
| --- | --- | --- |
| F1 | *Penicillium* sp. | MN861242 |
| F2 | *Talaromyces* sp. | MN861243 |
| F3 | *Acremonium* sp. | MN861244 |
| F4 | *Mortierella* sp. | MN861245 |
| F5 | *Monodictys* sp. | MN861246 |
| F6 | *Dactylonectria* sp. | MN861247 |
| F7 | *Penicillium* sp. | MN861248 |
| F8 | *Gliomastix* sp. | MN861249 |
| F9 | Nectriaceae sp. | MN861250 |
| F10 | *Dactylonectria* sp. | MN861251 |
| F11 | *Talaromyces* sp. | MN861252 |
| F12 | *Fusarium* sp. | MN861253 |
| F13 | *Dactylonectria* sp. | MN861254 |
| F14 | *Pyrenochaeta* sp. | MN861255 |
| F15 | *Plectosphaerella* sp. | MN861256 |
| F16 | *Dactylonectria* sp. | MN861257 |
| F17 | *Ilyonectria* sp. | MN861258 |
| F18 | *Dactylonectria* sp. | MN861259 |
| F19 | *Dactylonectria* sp. | MN861260 |
| F20 | *Penicillium* sp. | MN861261 |
| F21 | *Dactylonectria* sp. | MN861262 |
| F22 | *Cadophora* sp. | MN861263 |
| F23 | *Cadophora* sp. | MN861264 |
| F24 | *Cadophora* sp. | MN861265 |
| F25 | *Nectria* sp. | MN861266 |
| F26 | *Dactylonectria* sp. | MN861267 |
| F27 | *Leptodontidium* sp. | MN861268 |
| F28 | *Cylindrocladiella* sp. | MN861269 |
| F29 | *Ilyonectria* sp. | MN861270 |
| F30 | *Umbelopsis* sp. | MN861271 |
| F31 | *Penicillium* sp. | MN861272 |
| F32 | *Calonectria* sp. | MN861273 |
| F33 | *Cadophora* sp. | MN861274 |
| F34 | *Dactylonectria* sp. | MN861275 |
| F36 | *Dactylonectria* sp. | MN861276 |
| F37 | *Ilyonectria* sp. | MN861277 |
| F38 | *Penicillium* sp. | MN861278 |
| F39 | *Calonectria* sp. | MN861279 |
| F40 | *Ilyonectria* sp. | MN861280 |
| F41 | *Plectosphaerella* sp. | MN861281 |
| F42 | *Penicillium* sp. | MN861282 |
| F43 | *Penicillium* sp. | MN861283 |
| F44 | *Penicillium* sp. | MN861284 |
| F45 | *Penicillium* sp. | MN861285 |
| F46 | *Penicillium* sp. | MN861286 |
| F47 | *Penicillium* sp. | MN861287 |
| F48 | *Penicillium* sp. | MN861288 |
| F49 | *Penicillium* sp. | MN861289 |
| F50 | *Penicillium* sp. | MN861290 |
|  |  |  |
| RRD_1 | *Dactylonectria* sp. | MN861291 |
| RRD_2 | *Fusarium* sp. | MN861292 |
| RRD_3 | *Nectria* sp. | MN861293 |
| RRD_3a | *Nectria* sp. | MN861294 |
| RRD_5 | *Cadophora* sp. | MN861295 |
| RRD_7 | *Ilyonectria* sp. | MN861296 |
| RRD_8 | *Ilyonectria* sp. | MN861297 |
| RRD_10 | *Leptodontidium* sp. | MN861298 |
| RRD_11 | *Dactylonectria estremocensis* | MN861299 |
| RRD_12 | *Dactylonectria estremocensis* | MN861300 |
| RRD_13 | *Penicillium* sp. | MN861301 |
| RRD_14 | *Nectria* sp. | MN861302 |
| RRD_15 | *Fusarium* sp. | MN861303 |
| RRD_16 | *Nectria* sp. | MN861304 |
| RRD_17 | *Fusarium* sp. | MN861305 |
| RRD_19 | *Nectria* sp. | MN861306 |
| RRD_20 | *Dactylonectria* sp. | MN861307 |
| RRD_20a | *Dactylonectria* sp. | MN861308 |
| RRD_21 | *Ilyonectria* sp. | MN861309 |
| RRD_22 | *Fusarium* sp. | MN861310 |
| RRD_23 | *Nectria* sp. | MN861311 |
| RRD_24 | *Entrophospora* sp. | MN861312 |
| RRD_25 | *Fusarium* sp. | MN861313 |
| RRD_26 | *Nectria* sp. | MN861314 |
| RRD_27 | *Ilyonectria robusta* | MN861315 |
| RRD_28 | *Nectria* sp. | MN861316 |
| RRD_30 | *Ilyonectria* sp. | MN861317 |
| RRD_31 | *Dactylonectria* sp. | MN861318 |
| RRD_32 | *Dactylonectria* sp. | MN861319 |
| RRD_33 | *Fusarium* sp. | MN861320 |
| RRD_34 | *Fusarium* sp. | MN861321 |
| RRD_35 | *Fusarium* sp. | MN861322 |
| RRD_37 | *Nectria* sp. | MN861323 |
| RRD_42 | *Cladosporium* sp. | MN861324 |
| RRD_43 | *Fusarium* sp. | MN861325 |
| RRD_44 | *Nectria* sp. | MN861326 |
| RRD_45 | *Pilidium* sp. | MN861327 |
| RRD_47 | *Fusarium* sp. | MN861328 |
| RRD_48 | *Fusarium* sp. | MN861329 |
| RRD_50 | *Fusarium* sp. | MN861330 |
| RRD_51 | *Arthrobotrys* sp. | MN861331 |
| RRD_52 | *Nectria* sp. | MN861332 |
| RRD_53 | *Dactyloncetria* sp. | MN861333 |
| RRD_54 | *Ilyonectria* sp. | MN861334 |
| RRD_55 | *Calonectria* sp. | MN861335 |
| RRD_56 | *Fusarium* sp. | MN861336 |
| RRD_57 | *Fusarium* sp. | MN861337 |
| RRD_58 | *Dactylonectria pauciseptata* | MN861338 |
| RRD_59 | *Nectria* sp. | MN861339 |
| RRD_60 | *Nectria* sp. | MN861340 |
| RRD_62 | *Dactylonectria pauciseptata* | MN861341 |
| RRD_63 | *Ilyonectria* sp. | MN861342 |
| RRD_64 | *Nectria* sp. | MN861343 |
| RRD_65 | *Nectria* sp. | MN861344 |
| RRD_66 | *Fusarium* sp. | MN861345 |
| RRD_68 | *Robillarda* sp. | MN861346 |
| RRD_69 | *Nectria* sp. | MN861347 |
| RRD_70 | *Ilyonectria robusta* | MN861348 |
| RRD_71 | *Fusarium* sp. | MN861349 |
| RRD_72 | *Fusarium* sp. | MN861350 |
| RRD_73 | *Cylindrocladiella* sp. | MN861351 |
| RRD_74 | *Fusarium* sp. | MN861352 |
| RRD_75 | *Fusarium* sp. | MN861353 |
| RRD_76 | *Fusarium* sp. | MN861354 |
| RRD_77 | *Ilyonectria robusta* | MN861355 |
| RRD_78 | *Ceratobasidium* sp. | MN861356 |
|  |  |  |
| RRD_Oo-1 | *Pythium sylvaticum* | MN857689 |
| RRD_Oo-4 | *Pythium sylvaticum* | MN857691 |
| RRD_Oo-5 | *Pythium sylvaticum* | MN857692 |
| RRD_Oo-6 | *Pythium sylvaticum* | MN857693 |
| RRD_Oo-7 | *Pythium sylvaticum* | MN857694 |
| RRD_Oo-8 | *Pythium sylvaticum* | MN857695 |
| RRD_Oo-9 | *Pythium sylvaticum* | MN857696 |
| RRD_Oo-10 | *Pythium sylvaticum* | MN857697 |
| RRD_Oo-11 | *Pythium sylvaticum* | MN857698 |
| RRD_Oo-12 | *Pythium sylvaticum* | MN857699 |
| RRD_Oo-13 | *Pythium sylvaticum* | MN857700 |
| RRD_Oo-14 | *Pythium sylvaticum* | MN857701 |
| RRD_Oo-15 | *Pythium sylvaticum* | MN857702 |
| RRD_Oo-16 | *Pythium sylvaticum* | MN857703 |
| RRD_Oo-17 | *Pythium sylvaticum* | MN857704 |
| RRD_Oo-18 | *Pythium sylvaticum* | MN857705 |
| RRD_Oo-20 | *Phytopythium* sp. | MN857706 |
| RRD_Oo-21 | *Phytopythium* sp. | MN857707 |
