# Supplementary material for: Author Correction: Observations on early fungal infections with relevance for replant disease in fine roots of the rose rootstock Rosa corymbifera 'Laxa'
Source: Sci Rep. 2021 Aug 19;11:17172. doi: 10.1038/s41598-021-95182-5 (PMC8377036; doi:10.1038/s41598-021-95182-5)
Supplement: Supplementary file 1 — Supplementary Table 2. [file 41598_2021_95182_MOESM1_ESM.docx]

**Observations on early fungal infections with relevance for replant disease fine roots of the rose rootstock *Rosa corymbifera* 'Laxa'**

by G. Grunewaldt-Stöcker, C. Popp, A. Baumann, S. Fricke, M. Menssen, T. Winkelmann, E. Maiss.

**Table ESM 2** Identification of fungal root endophytes isolated from three *Rosa corymbifera* ‘Laxa’ plants nine weeks after cultivation in RRD Heidgraben soil. Sanger sequencing of genes Histone 3 (**HIS**), partial β-tubulin (**TUB**) and translation elongation factor 1-α (**TEF**) and internal transcribed spacer (**ITS**), first hits of BLASTn analysis

| **Isolate**  **Designation** | **Plant No.** | **Gene sequences used for identification** | | | |
| --- | --- | --- | --- | --- | --- |
|  |  | **HIS** | **TUB** | **TEF** | **ITS** |
| RRD 55 | 2 | *Calonectria montana* | *Calonectria canadiana* | failed^1^ | *Calonectria* sp. |
| RRD 53 | 2 | *Dactylonectria hordeicola* | *Cylindrocarpon obtusisporum* | *Ilyonectria* sp. | *Dactylonectria* sp. |
| RRD 58 | 2 | *Dactylonectria pauciseptata* | *Dactylonectria pauciseptata* | *Dactylonectria pauciseptata* | *Dactylonectria pauciseptata* |
| RRD 62 | 2 | *Dactylonectria pauciseptata* | *Dactylonectria pauciseptata* | *Dactylonectria pauciseptata* | *Dactylonectria pauciseptata* |
| RRD 54 | 2 | *Ilyonectria robusta* | *Ilyonectria robusta* | *Ilyonectria robusta* | *Ilyonectria* sp. |
| RRD 63 | 2 | *Ilyonectria robusta* | *Ilyonectria robusta* | *Ilyonectria robusta* | *Ilyonectria* sp. |
| RRD 65 | 2 | *Ilyonectria robusta* | *Ilyonectria robusta* | *Ilyonectria robusta* | *Nectria* sp. |
| RRD 52 | 2 | *Rugonectria rugulosa* | *Thelonectria rubrococca* | Hypocreales sp.^2^ | *Nectria* sp. |
| RRD 59 | 2 | *Rugonectria rugulosa* | *Thelonectria rubrococca* | Hypocreales sp. | *Nectria* sp. |
| RRD 60 | 2 | *Rugonectria rugulosa* | *Thelonectria* sp.^3^ | Hypocreales sp. | *Nectria* sp. |
| RRD 64 | 2 | *Rugonectria rugulosa* | *Thelonectria rubrococca* | Hypocreales sp. | *Nectria* sp. |
| RRD 73 | 3 | *Cylindrocladiella* sp. | *Cylindrocladiella* sp. | *Cylindrocladiella* sp. | *Cylindrocladiella* sp. |
| RRD 11 | 3 | *Dactylonectria estremocensis* | *Dactylonectria estremocensis* | *Dactylonectria estremocensis* | *Dactylonectria estremocensis* |
| RRD 12 | 3 | *Dactylonectria estremocensis* | *Dactylonectria estremocensis* | *Dactylonectria estremocensis* | *Dactylonectria estremocensis* |
| RRD 1 | 3 | *Dactylonectria torresensis* | *Dactylonectria torresensis* | *Dactylonectria torresensis* | *Dactylonectria* sp. |
| RRD 8 | 3 | *Dactylonectria torresensis* | *Dactylonectria torresensis* | *Dactylonectria torresensis* | *Ilyonectria* sp. |
| RRD 20 | 3 | *Dactylonectria torresensis* | *Dactylonectria torresensis* | *Dactylonectria torresensis* | *Dactylonectria* sp. |
| RRD 20a | 3 | *Dactylonectria torresensis* | *Dactylonectria torresensis* | *Dactylonectria torresensis* | *Dactylonectria* sp. |
| RRD 31 | 3 | *Dactylonectria torresensis* | *Dactylonectria torresensis* | *Dactylonectria torresensis* | *Dactylonectria* sp. |
| RRD 32 | 3 | *Dactylonectria torresensis* | *Dactylonectria torresensis* | *Dactylonectria torresensis* | *Dactylonectria* sp. |
| RRD 7 | 3 | *Ilyonectria robusta* | *Ilyonectria robusta* | *Ilyonectria robusta* | *Ilyonectria* sp. |
| RRD 27 | 3 | *Ilyonectria robusta* | *Ilyonectria robusta* | *Ilyonectria robusta* | *Ilyonectria robusta* |
| RRD 30 | 3 | *Ilyonectria robusta* | *Ilyonectria robusta* | *Ilyonectria robusta* | *Ilyonectria robusta* |
| RRD 70 | 3 | *Ilyonectria robusta* | *Ilyonectria robusta* | *Ilyonectria robusta* | *Ilyonectria robusta* |
| RRD 77 | 3 | *Ilyonectria robusta* | *Ilyonectria robusta* | *Ilyonectria robusta* | *Ilyonectria robusta* |
| RRD 3 | 3 | *Rugonectria rugulosa* | *Thelonectria* sp. | Hypocreales sp. | *Nectria* sp. |
| RRD 3a | 3 | *Rugonectria*  *rugulosa* | *Thelonectria* sp. | Hypocreales sp. | *Nectria* sp. |
| RRD 16 | 3 | *Rugonectria rugulosa* | *Thelonectria* sp. | Hypocreales sp. | *Nectria* sp. |
| RRD 19 | 3 | *Rugonectria rugulosa* | *Thelonectria* sp. | Hypocreales sp. | *Nectria* sp. |
| RRD 23 | 3 | *Rugonectria rugulosa* | *Thelonectria* sp. | Hypocreales sp. | *Nectria* sp. |
| RRD 26 | 3 | *Rugonectria rugulosa* | *Thelonectria rubrococca* | Hypocreales sp. | *Nectria* sp. |
| RRD 28 | 3 | *Rugonectria rugulosa* | *Thelonectria rubrococca* | Hypocreales sp. | *Nectria* sp. |
| RRD 37 | 3 | *Rugonectria rugulosa* | *Thelonectria rubrococca* | Hypocreales sp. | *Nectria* sp. |
| RRD 44 | 3 | *Rugonectria rugulosa* | *Thelonectria* sp. | Hypocreales sp. | *Nectria* sp. |
| RRD 69 | 1 | *Rugonectria rugulosa* | *Thelonectria rubrococca* | Hypocreales sp. | *Nectria* sp. |

^1^sequencing failed, ^2^Hypocreales sp. ICMP 13358 culture, ^3^first hit was *Coccinonectria pachysandricola*, second hit was *Thelonectria* sp.
